# The second polar body contributes to the fate asymmetry in the mouse embryo

**DOI:** 10.1093/nsr/nwac003

**Published:** 2022-01-10

**Authors:** Hongbin Jin, Yang Han, Huasong Wang, J Xiao He Li, Weimin Shen, Lin Zhang, Luxi Chen, Shunji Jia, Ping Yuan, Hui Chen, Anming Meng

**Affiliations:** Laboratory of Molecular Developmental Biology, State Key Laboratory of Membrane Biology, Tsinghua-Peking Center for Life Sciences, School of Life Sciences, Tsinghua University, Beijing 100084, China; Laboratory of Molecular Developmental Biology, State Key Laboratory of Membrane Biology, Tsinghua-Peking Center for Life Sciences, School of Life Sciences, Tsinghua University, Beijing 100084, China; Laboratory of Molecular Developmental Biology, State Key Laboratory of Membrane Biology, Tsinghua-Peking Center for Life Sciences, School of Life Sciences, Tsinghua University, Beijing 100084, China; Laboratory of Molecular Developmental Biology, State Key Laboratory of Membrane Biology, Tsinghua-Peking Center for Life Sciences, School of Life Sciences, Tsinghua University, Beijing 100084, China; Laboratory of Molecular Developmental Biology, State Key Laboratory of Membrane Biology, Tsinghua-Peking Center for Life Sciences, School of Life Sciences, Tsinghua University, Beijing 100084, China; Laboratory of Molecular Developmental Biology, State Key Laboratory of Membrane Biology, Tsinghua-Peking Center for Life Sciences, School of Life Sciences, Tsinghua University, Beijing 100084, China; Laboratory of Molecular Developmental Biology, State Key Laboratory of Membrane Biology, Tsinghua-Peking Center for Life Sciences, School of Life Sciences, Tsinghua University, Beijing 100084, China; Laboratory of Molecular Developmental Biology, State Key Laboratory of Membrane Biology, Tsinghua-Peking Center for Life Sciences, School of Life Sciences, Tsinghua University, Beijing 100084, China; Center for Reproductive Genetics and Reproductive Medicine, Sun Yat-sen Memorial Hospital, Sun Yat-sen University, Guangzhou 510120, China; Center for Reproductive Genetics and Reproductive Medicine, Sun Yat-sen Memorial Hospital, Sun Yat-sen University, Guangzhou 510120, China; Laboratory of Molecular Developmental Biology, State Key Laboratory of Membrane Biology, Tsinghua-Peking Center for Life Sciences, School of Life Sciences, Tsinghua University, Beijing 100084, China; Center for Reproductive Genetics and Reproductive Medicine, Sun Yat-sen Memorial Hospital, Sun Yat-sen University, Guangzhou 510120, China; Guangzhou Laboratory, Guangzhou 510320, China

**Keywords:** mouse, embryo, polar body, cell fate, asymmetry

## Abstract

The polar bodies (PBs) are extruded microcells during oocyte meiosis and generally regarded as inessentials for embryonic development. Therefore, PBs have been widely used as important materials for pre-implantation genetic diagnosis in human. Here we report that the second PB (PB2) in the mouse zygote may play roles in cell-fate specification and post-implantation development. A subset of mRNAs encoding pluripotency-related factors are enriched in PB2. Nascent proteins may be synthesized in PB2 after fertilization and transport from PB2 to the zygote before the two-cell stage. The PB2-attached blastomere (pbB) at the two-cell stage, compared to the other blastomere (npbB), likely contributes more descendants to the inner cell mass (ICM) lineage in the blastocyst. Removal of PB2 from the zygote or transient blockage of material exchange between PB2 and the zygote by nocodazole treatment appears to cause a loss of the ICM fate bias of pbB. PB2 removal or nocodazole treatment also results in abnormal post-implantation development. Injection of PB2 lysate into pbB of PB2-removed two-cell-stage embryos may reset the cell-fate preference and rescue post-implantation development. Our data collectively suggest that PB2 would demarcate the earliest cell-fate asymmetry of the mouse zygote and be required for post-implantation development.

## Teaser

The second polar body marks the asymmetry of the mouse zygote and plays important role in regulating pre-implantation fate determination as well as post-implantation embryonic development.

## INTRODUCTION

How an animal embryo of multiple cells starts to differentiate spatially into distinct cell fates is a fundamental question in biology. In non-mammalian species such as fly, fish and frog, unequal distribution of maternal factors in the oocyte plays a key role in the symmetry breaking of embryonic cells and establishment of the body axes [1–5]. In mammalian species, however, it remains unknown whether there exist asymmetrically distributed maternal factors predicting future body axes. In the mouse embryo, apparent asymmetry is morphologically visible in the cavitated blastocyst, the inner cell mass (ICM) of which will give rise to the embryo proper and the outer epithelial layer of which is the trophectoderm with the placenta fate [6,7]. The asymmetry of the blastocyst may be traced back to cell polarity and asymmetric cell division at the 8- to 16-cell stages [8–10], asymmetric nuclear abundance of Carm1 at the four-cell stage [11] and even further back to asymmetric expression of the long non-coding RNA *LincGET* between two blastomeres at the two-cell stage [12]. It is unclear whether it is the asymmetry of the mammalian zygote that determines the asymmetric fates of blastomeres during early development.

At the last step of mammalian follicle maturation, the mature oocyte (egg) extrudes the first polar body (PB1) that contains a diploid nucleus and little cytoplasm. Following fertilization, the egg nucleus resumes the second meiotic division, resulting in elimination of half of the chromosomes via the extrusion of the second polar body (PB2). The mouse PB1 degenerates soon after extrusion [13], whereas PB2 may survive for 3 days [14–16]. It is widely accepted that the mammalian PB1 and PB2 are not required for embryonic development [17–20]. Thus, polar body biopsy has been applied for pre-implantation genetic testing to select normal embryos in human assisted reproduction [21,22]. However, some earlier studies suggest that the survived PB2 in the early blastocyst may be a marker of the blastocyst bilateral symmetry axis orthogonal to the embryonic-abembryonic axis and the ICM cells closer to the PB2 in the blastocyst are more likely to become distal visceral endodermal cells in the post-implantation embryo [16,23,24]. Therefore, we decided to reinvestigate the necessity of PB2 for asymmetric fate establishment of the mouse pre-implantation embryo as well as for post-implantation development.

## RESULTS

### PB2 may possess translation and transcription activity

**Figure 1.**
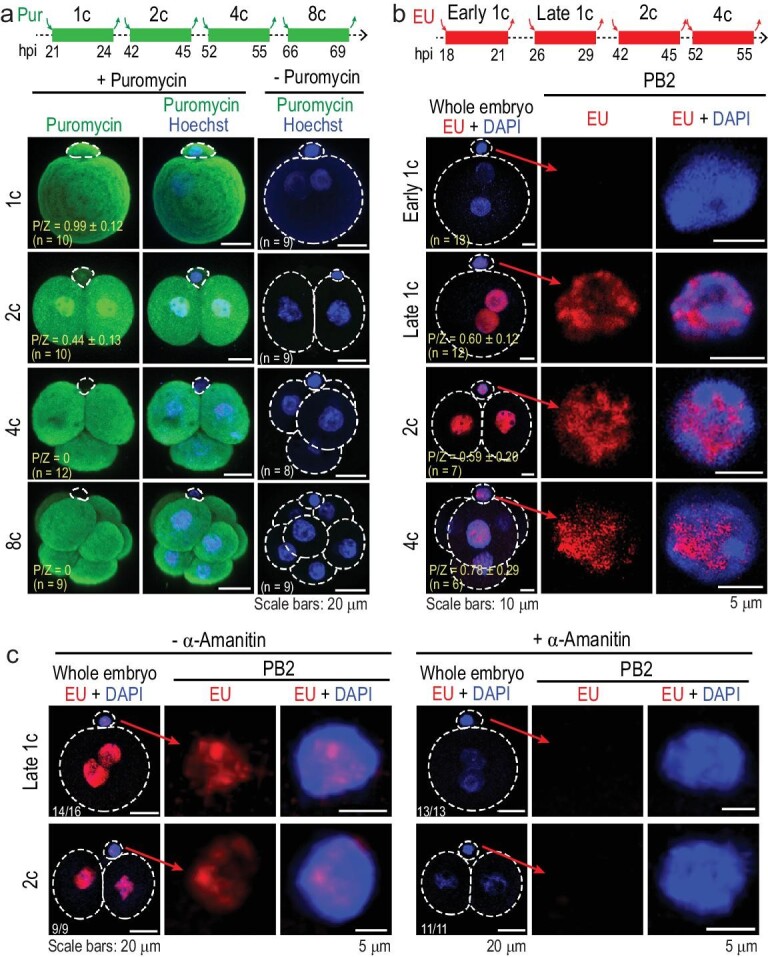
Translation and transcription activity in PB2. (a) Translation activity revealed by immunostaining with puromycin antibody. Embryos at an indicated stage were incubated in the presence of puromycin for 3 h. The top illustration shows the treated period referred to hours post human chorionic gonadotrophin (HCG) injection (hpi). The right column: negative controls with no signal. P/Z, ratio of mean signal intensity in PB2 to that in the zygote; *n*, number of observed embryos (same in (b)). (b) Transcription activity revealed by EU incorporation assay. Embryos at an indicated stage were incubated in the presence of EU for 3 h as indicated on the top illustration. (c) Inhibition of transcription activity by α-amanitin. One-cell stage embryos were incubated in the presence of α-amanitin throughout but with the addition of EU at different stages for the indicated duration, followed by EU staining. All images were acquired by confocal microscopy. The ratio of observed embryos with the representative pattern is indicated.

Compared to the zygote, PB2 is a specialized tiny cell body with a little ooplasm and so may not contain the necessary translation and transcription machineries. However, our immunostaining assays revealed the presence of 28S/18S ribosomal RNAs, RNA polymerase II and active (phospho-Ser5) RNA polymerase II in PB2 at the one-cell stage (Supplementary Fig. S1), suggesting the existence of translation and transcription machineries. Then, we performed puromycin incorporation assays by culturing embryos in the presence of puromycin for 3 h within individual cell cycles from the one-cell to eight-cell stages. Subsequent immunostaining detected strong puromycin signal in blastomeres at all tested stages as well as in PB2 at the one-cell (strong) and two-cell (weaker) stages but not at later stages (Fig. 1a), indicating translation activity of PB2 from fertilization to the two-cell stage. To detect nascent RNAs, we performed an EU (analogue of uridine 5′-triphosphate (UTP)) incorporation experiment. Results indicated that active transcription likely occurred in PB2 at least from the late one-cell to four-cell stages (Fig. 1b and c).

### Exogenous proteins may transport between PB2 and the zygote

**Figure 2.**
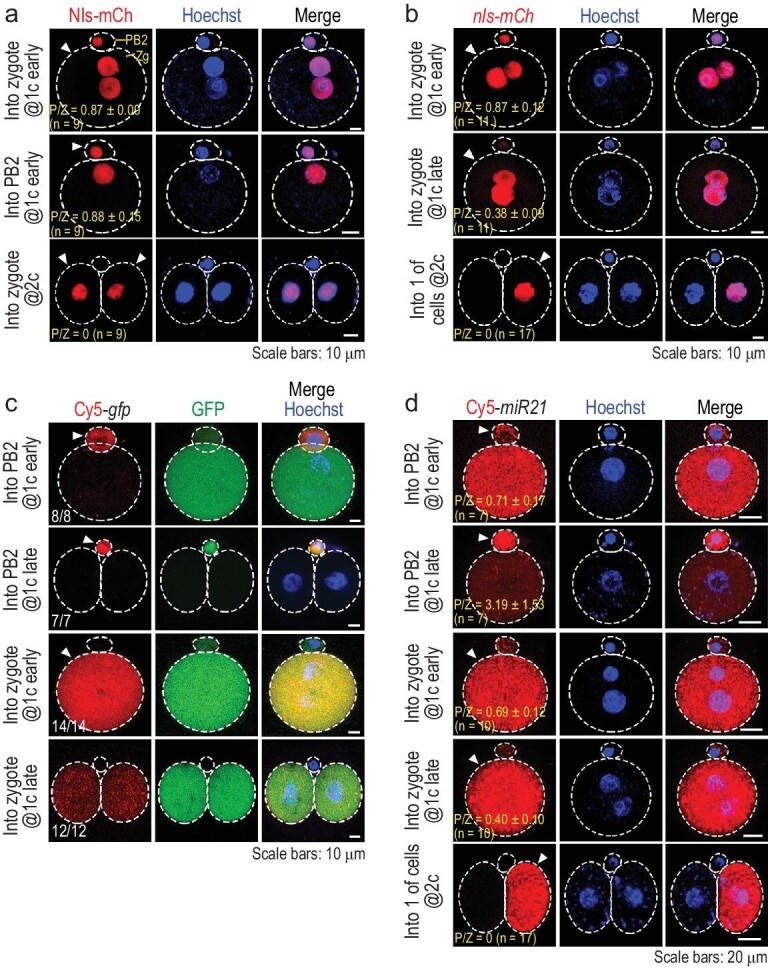
Protein and RNA transport between PB2 and the zygote. The embryos were injected at an indicated stage and directly observed by confocal microscopy ∼4 h post injection. The injected cell is indicated by an arrowhead. DNA/nucleus was stained by Hoechst 33342. The ratio of observed embryos with the representative pattern is indicated at the bottom left corner. Zg, zygote. (a) Transport of injected Nls-mCherry protein. (b) Transport of Nls-mCherry following *nls-mCherry* mRNA. (c) Transport of Cy5-labeled full-length *gfp* mRNA. The ratio of embryos with the representative pattern is indicated. (d) Transport of Cy5-labeled *miR21-3p* RNA. P/Z, ratio of mean signal intensity in PB2 to that in the zygote (a), (b) and (d); *n*, the number of observed embryos.

The physical connection between PB2 and the zygote suggests a possibility of material exchange between them. Then, we injected the purified nuclear localization signal (Nls)-mCherry protein into PB2 or the zygote/blastomeres at the one- or two-cell stage. Results showed that the one-cell stage injection led to strong Nls-mCherry fluorescence not only in the injected cell, but also in the un-injected cell (Fig. 2a), whereas Nls-mCherry injected into both blastomeres at the two-cell stage did not produce fluorescence in PB2. These results suggest that proteins could transfer bidirectionally between PB2 and the zygote during the first cleavage period and this transfer most likely ceases by the two-cell stage. Fluorescent microscopy revealed that Nls-mCherry injected into PB2 at the one-cell stage visibly flowed into the zygote within 1 s; but Nls-mCherry injected into the zygote became visible in PB2 about 1 min after injection (Supplementary Video S1). It appears that proteins would transport from PB2 to the zygote faster than from the zygote to PB2.

### Small RNAs could transport between PB2 and the zygote

Next, we tested whether RNA could transfer between PB2 and the zygote. First, we observed that injection of *nls-mCherry* mRNA into the zygote at the early or late one-cell stage also resulted in a strong or weak fluorescence signal soon in PB2, respectively, whereas injection into the zygote at the late two-cell stage led to no fluorescence in PB2 (Fig. 2b). Then, Cy5-labeled full-length *gfp* mRNA (710 nucleotides (nt)) was injected into PB2 or the zygote at the early or late one-cell stage. Results showed that, regardless of the injection stage, Cy5 signal existed only in the injected cell but was hardly seen in the un-injected cell(s) (Fig. 2c), suggesting that *gfp* mRNA should be unable to transfer between PB2 and the zygote. Notably, GFP protein translated from Cy5-*gfp* mRNA injected into PB2 was seen in PB2, supporting the notion that PB2 has the translation activity. When the RNA size was reduced to 100–500 nt, a size-dependent zygote-to-PB2 transport efficiency was observed (Supplementary Fig. S2a and b). Then, we tested *miR21-3p*, a 22-nt microRNA. We found that bidirectional transportation of Cy5-*miR21-3p* between PB2 and the zygote mainly happened at the early one-cell stage, became weaker at the late one-cell stage and did not happen if injected at the two-cell stage (Fig. 2d). Besides, transportation of injected FITC-labeled morpholino oligonucleotide (25-nt) behaved similarly (Supplementary Fig. S2c). Thus, transportability of RNAs between PB2 and the zygote might largely depend on the RNA size and developmental stage.

### Protein and RNA transport may depend on the mid-body

Microtubules, the tubulin polymers, play important roles in many cellular processes including cell division and macromolecules and vesicles transportation [25]. During extrusion of PB2 in the mouse zygote, the meiotic spindle-derived mid-body with bundles of microtubules forms to transiently connect PB2 and the zygote [26]. To test the possible implication of the mid-body in the PB2–zygote exchange of proteins and RNAs, we treated early zygotes for 3 h with the microtubule inhibitor nocodazole to disrupt the mid-body. The mid-body was absent after nocodazole treatment and could not be restored 4 h post-wash when microtubules in the cytoplasm of the zygote were reforming (Supplementary Fig. S3a and b). When early zygotes injected with Nls-mCherry protein or Cy5-*miR21-5p* RNA were incubated similarly in the presence of nocodazole, either of the injected materials failed to transport (Supplementary Fig. S3c). Therefore, protein and RNA transport between PB2 and the zygote may mainly rely on the mid-body between them.

### The PB2-attached blastomere may have an ICM fate preference

During mitosis of the mouse zygote, the first cleavage furrow usually forms apart from the PB2-attached region [27], implying that PB2 is directly connected to only one daughter blastomere (pbB) but distant from the other blastomere (npbB). However, PB2 may move away from the original position on the zygote surface near and after the completion of the first cleavage [28] (see also Supplementary Fig. S4a and c), causing the uncertainty to faithfully recognize the pbB and its descendants later on. We found that an olive oil droplet injected into the area underneath PB2 (PB side) or into the opposite area (NPB side) within the zygote appeared stationary during the first cleavage and was almost entirely left in pbB or npbB at the two-cell stage, respectively (Supplementary Fig. S4b and c, and Supplementary Video S2). Thus, the injected oil droplet would allow easy identification of two different blastomeres.

**Figure 3.**
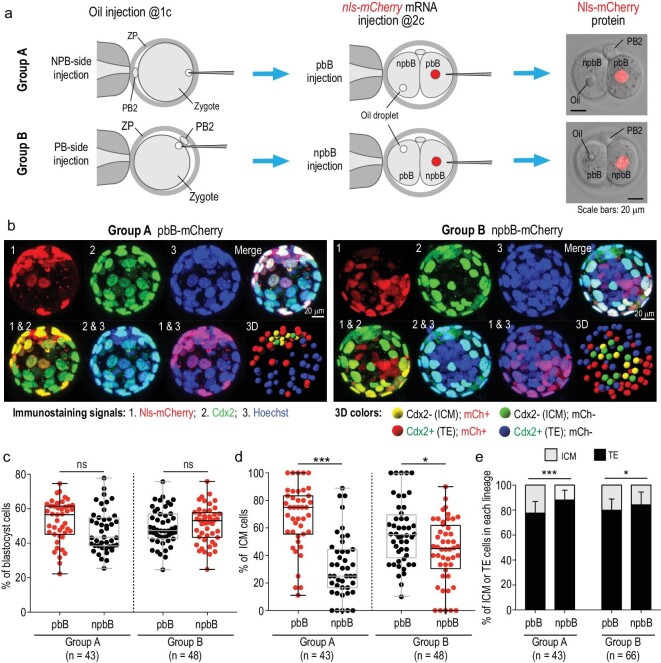
pbB descendants may contribute more to the ICM. (a) Two injection strategies. ZP, zona pellucida; pbB, polar body-attached blastomere; npbB, blastomere opposite to pbB. Right column: Nls-mCherry protein expressed from injected *nls-mCherry* mRNA. (b) Example of Z-stack confocal and 3D-reconstituted images of blastocysts after immunostaining with mCherry and Cdx2 antibodies together with Hoechst staining. The inner cells were ascertained by viewing Z-slices and 3D images from different angles (see also Supplementary Video S3). (c) and (d) Percentage of pbB- or npbB-derived blastocyst cells (c) or ICM cells (d). Each dot represents the percentage from one blastocyst. Box and whiskers plot (c) and (d), from minimum to maximum with the median indicated by the center line; box limits, upper and lower quartiles; *n*, number of observed blastocysts. (e) Proportion of ICM or TE cells derived from pbB or npbB. Statistical significance: ns, non-significant (*P* > 0.05); ^*^P < 0.05; ^***^P < 0.001.

We then traced the lineages of pbB’s or npbB’s descendants in several ways during early development based on oil labeling. First, an oil droplet was injected into the NPB (Group A) or the PB side (Group B) at the one-cell stage and then with a second injection of *nls-mCherry* mRNA into the blastomere without oil droplet at the late two-cell stage (Fig. 3a). The injected embryos were immunostained at blastocyst stages against mCherry and the trophectoderm (TE) marker Cdx2 together with Hoechst 33342 staining for DNA, and the Cdx2-negative (Cdx2^–^) inner cells were recognized by viewing confocal Z-slices and a 3D-reconstituted image of each embryo (Fig. 3b and Supplementary Video S3). We found that, in either injection way, the average percentage of pbB descendants in the blastocyst appeared comparable to that of npbB descendants (Fig. 3c); however, 67.8% or 56.7% of ICM cells were derived from pbB without oil or with oil, respectively, both being higher than the rate of npbB-derived ICM cells (Fig. 3d). It appeared that the oil droplet underneath PB2 might slightly interfere in the pbB differentiation into the ICM possibly due to the adverse effect on the PB2-to-zygote information transfer, and so we chose to inject oil into the NPB side in subsequent assays. In another calculating way, the chance of a pbB descendant becoming the ICM also appeared higher than that of npbB descendants (Fig. 3e). Even if both the oil droplet and *nls-mCherry* mRNA were injected into npbB at the two-cell stage, a higher proportion of Cdx2^–^ inner cells in blastocysts were derived from pbB (Supplementary Fig. S5). In contrast, when *nls-mCherry* mRNA was randomly injected into one of the blastomeres at the two-cell stage, 51.2% of the ICM cells were Nls-mCherry-positive (Supplementary Fig. S6), being unable to disclose biased contributions of different blastomeres. Second, by immunostaining injected embryos for the ICM marker Sox2 or the TE marker active Yap1, we also identified more pbB-derived Sox2-positive or Yap1-negative ICM cells (Supplementary Fig. S7). Third, we observed live embryos by confocal microscopy after injection with *CAAX-gfp* (labeling plasma membrane) and *nls-mCherry* (labeling pbB) mRNAs and identified inner cells based on their locations. Result indicated that Nls-mCherry-labeled descendants of pbB might contribute more to the ICM (Supplementary Fig. S8 and Supplementary Video S4). Finally, developmental potential test of two-blastomere reaggregates disclosed that npbB/npbB reaggregates developed with a lower blastocyst rate and that npbB/npbB- or pbB/pbB-derived blastocysts had fewer inner or outer cells than pbB/npbB-derived blastocysts (Supplementary Fig. S9), which suggests that normal pre-implantation development might rely on the co-existence of pbB and npbB. These data collectively support the idea that attachment of PB2 may demarcate the zygote asymmetry and preset pbB for the ICM lineage preference.

### PB2 removal may affect pre-implantation fate bias and post-implantation development

**Figure 4.**
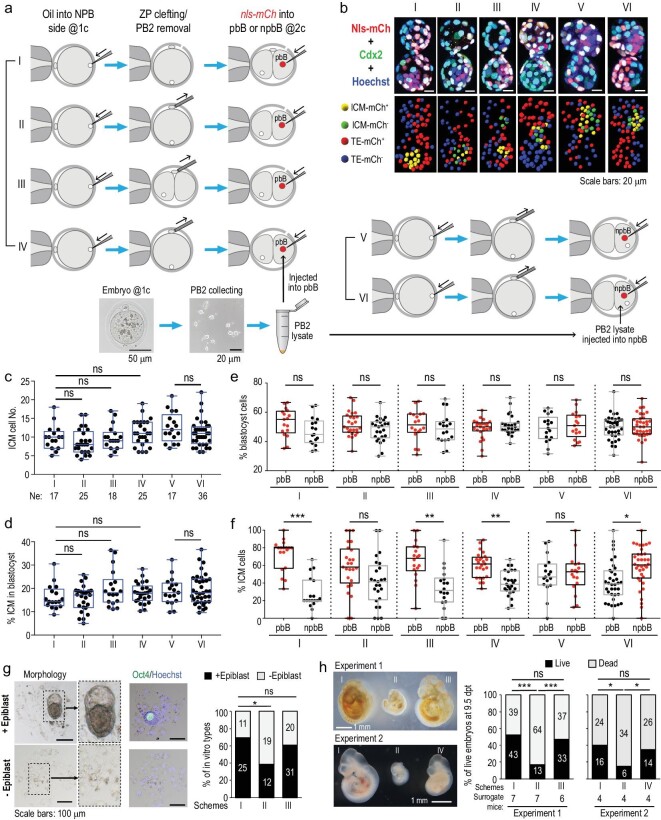
Effect of PB2 removal on pre-implantation cell-fate specification. (a) Schemes of manipulations. (b) Example of confocal images of blastocysts after immunostaining with mCherry and Cdx2 antibodies together with Hoechst staining. Note that blastocysts hatched earlier due to the hole in ZP. (c)–(f) Box and whiskers plots showing total number of ICM cells (c), percentage of ICM cells in the blastocyst (d) and percentage of pbB- or npbB-derived blastocyst cells (e) or ICM cells (f). Each dot represents the percentage from one blastocyst. (g) Effect of PB2 removal on *in vitro* post-implantation development. Embryos *in vitro* cultured to 7.5 days post fertilization were observed for morphology (left) and for Oct4 expression (middle). The percentage of embryos with an epiblast-like structure was calculated. (h) Effect of PB2 removal on *in vivo* post-implantation development. Embryos treated as shown in (a) were transferred to surrogate mice and the percentage of live embryos with normal tissues at 9.5 days post transplantation was calculated (right panel). Morphology of representative embryos is shown in the left panels. The dead embryos included non-implanted blastulas as well as implanted but deformed embryos. In the bar graphs, the total embryo numbers (pooled from several batches) are shown within the bar areas. Statistical significance: ns, non-significant (*P* > 0.05); ^*^P < 0.05; ^**^P < 0.01; ^***^P < 0.001.

To test whether material exchange between PB2 and the zygote is implicated in the ICM fate preference of pbB descendants, we first investigated the effect of PB2 removal on the cell fate. For all manipulations, an oil droplet was injected into the NPB side within the zygote at the one-cell stage, and PB2 was then removed at the middle one-cell (ΔPB2^@1C^) or two-cell stage (ΔPB2^@2C^) (Supplementary Fig. S10 and Supplementary Video S5), followed by injection of *nls-mCherry* mRNA into pbB (Fig. 4a, Schemes II and III). For control embryos (Scheme I), a cleft was made in the zona pellucida (ZP). Embryos without PB2 looked morphologically normal, with two-cell and blastocyst survival rates comparable to untreated embryos (Supplementary Fig. S11), which is consistent with a previous report [18]. The manipulated embryos, which hatched earlier due to the presence of a hole in the ZP, were fixed at the blastocyst stage for immunostaining against mCherry and Cdx2, and observed by confocal microscopy (Fig. 4b). Results showed that PB2 removal at either stage did not significantly alter the total number of ICM cells (Fig. 4c), the percentage of ICM cells (Fig. 4d) or the overall proportion of pbB or npbB descendants in blastocysts (Fig. 4e). However, the average percentage of pbB-ΔPB2^@1C^ descendants in the ICM appeared comparable to that of npbB descendants, while that of pbB-ΔPB2^@2C^ descendants in the ICM was still higher than that of npbB (Fig. 4f). This result suggests that removal of PB2 at the one-cell stage should gives rise to random fate choice of pbB and npbB. Importantly, the pbB-ΔPB2^@1C^ ICM fate preference in blastocysts could be restored when PB2 lysate obtained from PB2s at the late one-cell stage was injected into pbB-ΔPB2^@1C^ at the two-cell stage (Scheme IV, Fig. 4f). If PB2 lysate was injected into npbB of two-cell stage ΔPB2^@1C^ embryos (Scheme VI), npbB descendants accounted for a higher proportion of ICM cells than pbB descendants (Fig. 4f), suggesting resetting of the ICM fate preference of the pbB. These data together suggest that two blastomeres of the mouse two-cell stage embryo may acquire asymmetric cell fates and PB2 probably contains signals for promoting the ICM cell fate of pbB.

Given that blastocysts developed from PB2-removed zygotes look morphologically normal, we wondered whether they undergo normal post-implantation development. Through *in vitro* culture until 7.5 days post fertilization (∼E5.5), we found that the proportion of embryos with a distinguishable epiblast-like structure was 69.4% for the control embryos with a cleft, 60.8% for ΔPB2^@2C^ embryos and 38.7% for ΔPB2^@1C^ embryos, while the other embryos failed to form a cell mass with visible structure (Fig. 4g). Then, we transplanted morulae at four-cell to eight-cell stages into a single oviduct of pseudo-pregnant female mice. After failure to obtain any pups from several surrogate mice receiving ΔPB2^@1C^ morulae, we decided to dissect surrogate mice at 9 days post transplantation. We calculated live embryo rate (number of live embryos/total number of transplanted morulae). Results showed that the live embryo rate was 52.4%, 47.1% and 16.9% for control morulae with a cleft, ΔPB2^@2C^ morulae and ΔPB2^@1C^ morulae (Fig. 4h, right panel), respectively. Besides, all of the live ΔPB2^@1C^-derived embryos looked much smaller or abnormal in other aspects (Fig. 4h, left panel). The observations that ΔPB2^@1C^-derived blastocysts have ICM/TE cell numbers comparable to wild-type embryos but with different cell composition (Fig. 3c–e and Fig. 4c–f) and undergo abnormal post-implantation development (Fig. 4g and h) suggest that pbB-derived ICM/TE cells likely differ in intrinsic activity from npbB-derived cells. Importantly, ΔPB2^@1C^ embryos injected with PB2 lysate gave rise to a live embryo rate comparable to that of control embryos with a cleft after transplantation (Fig. 4h), demonstrating a potential rescue effect. Thus, PB2 may play an important role in post-implantation development.

### Blockage of PB2–zygote material exchange may affect pre-implantation fate specification and post-implantation development

As described above, protein and RNA transport between PB2 and the zygote could be blocked by transient nocodazole treatment (Supplementary Fig. S3c). We wondered whether such a non-invasive treatment could also affect the pre-implantation cell-fate determination and post-implantation development. As shown in Supplementary Fig. S12, the total cell number per blastocyst was comparable between control and treated blastocysts (Supplementary Fig. S12c); however, compared to control blastocysts with more ICM cells from pbB, blastocysts derived from nocodazole-treated zygotes had a similar number of ICM cells from pbB and npbB (Supplementary Fig. S12d). This observation suggests that material exchange between PB2 and the zygote should be important for establishing pre-implantation cell-fate asymmetry. When cultured *in vitro*, the rate of embryos with epiblast-like structure in nocodazole group was significantly lower than that in the control (DMSO-treated) group (Supplementary Fig. S12e). When transferred into surrogate mice, the rate of live embryos (all being much smaller) in the nocodazole group was much lower than that in the control group (Supplementary Fig. S12f). These results further support the idea that the PB2–zygote material exchange may be required for post-implantation development.

### PB2 may regulate embryonic development by providing pluripotency-related signaling molecules

**Figure 5.**
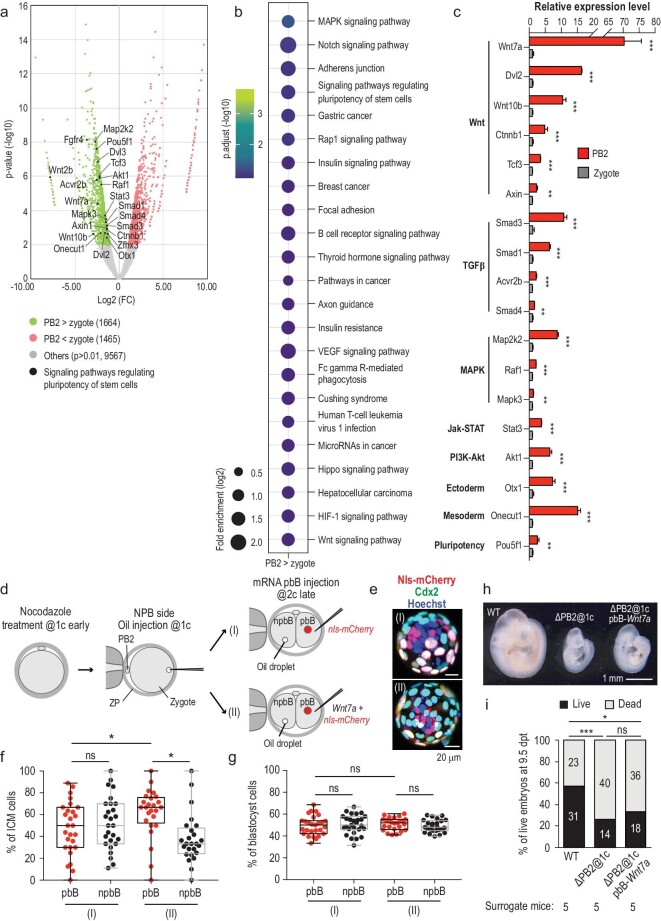
Identification of potential regulators provided by PB2. (a) Volcano plot of differentially expressed genes between PB2 and the zygote at the one-cell stage. The analysis was based on RNA-seq data of PB2 and the zygote. Some genes involved in the pluripotency of stem cells were upregulated in PB2 and marked by black dots in the plot. (b) Kyoto Encyclopedia of Genes and Genomes (KEGG) enrichment analysis of genes highly expressed in PB2. (c) RT-qPCR analysis of some genes that were enriched in PB2 and related to signaling pathways regulating the pluripotency of stem cells. (d) Illustration of manipulations for testing Wnt7a overexpression effect. Embryos were treated with nocodazole for 3 h at the early one-cell stage. Then oil was injected to label the NPB side of the embryos. *Wnt7a-HA* mRNA was injected into pbB together with *nls-mCherry* mRNA at the late two-cell stage (II). For the control group, *nls-mCherry* mRNA was injected into pbB (I). (e) Examples of immunostained blastocysts in each group. (f) and (g) Percentage of pbB- or npbB-derived ICM cells (f) or blastocyst cells (g). Each dot represents the percentage from one blastocyst. (h) Morphology of representative live embryos dissected from surrogate mice. Wild-type (WT) embryos with PB2 removed at the one-cell stage (ΔPB2@1c) or ΔPB2@1c embryos with Wnt7A mRNA injection into pbB at the two-cell stage were transferred to surrogate mice and the mice were dissected at 9.5 days post transplantation. (i) Percentage of live embryos with normal tissues and dead embryos (non-implanted or deformed) at. 9.5 days post transplantation in each group. Statistical significance: ns, non-significant (*P* > 0.05); ^*^P < 0.05; ^**^P < 0.01; ^***^P < 0.001.

As the above data suggest potential roles of PB2 in embryonic development, we set out to look for important molecules present in PB2. Our RNA-seq data from PB2 and the zygote revealed that 99.67% (12 573/12 615) of genes expressed in PB2 were also expressed in the zygote. There were 1664 genes upregulated in PB2, which included those involved in multiple signaling pathways (Fig. 5a and b). By RT-qPCR analysis, we confirmed enhanced expression levels of a set of pluripotency-related genes in PB2, including Wnt, transforming growth factor β (TGF-β) and mitogen-activated protein kinase (MAPK)-pathway components (Fig. 5c). Among those, Wnt7a attracted our attention due to its prominent enrichment in PB2 and implication of Wnt signaling in mouse early embryogenesis [29,30]. We found that overexpression of *Wnt7a-HA* mRNA in pbB of nocodazole-pretreated two-cell stage embryos led to regaining of the pbB descendants’ ICM fate preference (Fig. 5d–g); when transferred to surrogate mice, overexpression of *Wnt7a-HA* mRNA in pbB of PB2-removed embryos generated only smaller live embryos with a rate of 33.3% (18/54) at 9.5 days post transplantation, which was not significantly higher than the rate (25.9% or 14/54) for PB2-removed blastulas (Fig. 5h and i). The Wnt7a failure to rescue post-implantation development is likely ascribed to the inappropriate activity level and duration of ectopic Wnt7a or to the lack of other factors enriched in PB2.

## DISCUSSION

**Figure 6.**
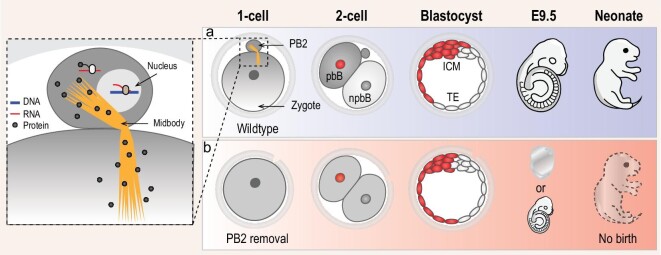
Model of PB2 function. (a) Normal developmental process of the zygote with PB2. Proteins translated from mRNAs in PB2 may transport to the zygote and probably form gradients along the animal–vegetal axis, resulting in higher amounts in pbB and lower amounts in npbB at the two-cell stage. Then, pbB may give rise to more ICM cells than npbB in the blastocyst that would develop normally. (b) Abnormal development of the zygote without PB2. Following PB2 removal, the zygote does not receive PB2-derived proteins, probably resulting in loss of asymmetry between pbB and npbB. In this scenario, the blastocyst looks morphologically normal but the ICM consists of a comparable number of pbB and npbB descendants; this blastocyst may undergo implantation failure or embryonic death, or form a small fetus that cannot survive to birth.

It is well accepted that PB2 extrusion from the mammalian oocyte upon fertilization aims to ensure the generation of haploid female pronucleus without direct contribution to embryonic development. In this study, we have demonstrated that PB2 in the mouse zygote may help to break the symmetry of the zygote through PB2–zygote material exchange, endowing pbB with the ICM lineage preference (Fig. 6).

We have demonstrated that the mouse PB2 might have retained translation and transcription activities. Although bidirectional material exchange happens between PB2 and the zygote during the first cleavage period, the PB2-to-zygote transport is likely to be more important for embryonic development because this directional transport is faster (Supplementary Video S1) and PB2 removal has an obvious adverse effect that could be rescued by PB2 lysate (Fig. 4). Given that the tiny size of PB2 would not allow storage of large amounts of proteins, it is most likely to be PB2-containing mRNAs that each may synthesize thousands of protein molecules within a short time for transporting to and functioning in the zygote. We hypothesize that some PB2-derived proteins may form gradients in the zygote through special mechanisms, which then would asymmetrically pass on to daughter blastomeres and cause the cell-fate bias. We have identified many PB2-enriched gene transcripts and some of them are related to cell pluripotency (Fig. 5a–c). Development of approaches for blastomere-specific depletion and controllable overexpression of those genes will be needed to investigate their functions and underlying mechanisms in the future.

In our study, we adopted two approaches to disrupt the potential function of PB2, i.e. physical removal of PB2 and transient nocodazole treatment of the fertilized egg. The former handling might result in the leakage of some cytoplasmic components of the zygote and the effect of mechanical stress, while the nocodazole treatment might exert other unknown, nonspecific effects. To confirm PB2 function, therefore, other approaches could be sought to disrupt PB2 bioactivity in the future, e.g. by laser inactivation of PB2.

We performed the above experiments in the mouse inbred strain C57BL/6N. To exclude the possibility of strain-specific PB2 function, we briefly repeated some experiments in the mouse ICR outbred strain. Results also confirmed the PB2–zygote protein/RNA transport feature and the ICM fate bias of the pbB (Supplementary Fig. S13). However, we currently do not know whether PB2 function is conserved in other mammalian species, in particular in human.

## METHODS

Detailed materials and methods are available in the Supplementary Data.

## Data availability

The RNA-seq data have been deposited on the NCBI database with an accession number GSE182918. The other data are available within the article and supplementary information files.

## Supplementary Material

nwac003_Supplemental_Files
