# Supplementary material for: The second polar body contributes to the fate asymmetry in the mouse embryo
Source: Natl Sci Rev. 2022 Jan 10;9(7):nwac003. doi: 10.1093/nsr/nwac003 (PMC9337984; doi:10.1093/nsr/nwac003)
Supplement: nwac003_Supplemental_Files [file nwac003_supplemental_files.zip › Supplementary_Data.docx]

# SUPPLEMENTARY INFORMATION FOR

**The second polar body contributes to the fate asymmetry in the mouse embryo**

Hongbin Jin^1,4^, Yang Han^1,4^, Huasong Wang^1^, J. Xiao He Li^1^, Weimin Shen^1^, Lin Zhang^1^, Luxi Chen^1^, Shunji Jia^1^, Ping Yuan^2^, Hui Chen^2^, Anming Meng^1,2,3^*

Correspondence: Anming Meng (mengam@mail.tsinghua.edu.cn)

**This PDF file includes:**

Methods

Supplementary Table S1

Supplementary Figures S1-S13

Captions for Supplementary Movies S1-S5

METHODS

**Mouse embryo collection**

The mouse strain C57BL/6N were usually used until otherwise stated (the mouse ICR outbred strain for Fig. S13). Mice were maintained on a 12/12-hour light/dark cycle, 22-26℃ with sterile pellet food and water ad libitum. 12-14 g female mice were super-ovulated by intraperitoneal injection of 10 IU pregnant mare serum gonadotropin (PMSG, Sansheng Biological Technology, 21958956) and 10 IU human chorionic gonadotrophin (HCG, Sansheng Biological Technology, 110041282) at the interval of 47.5 h. Then, the female mice were mated with 8 to 20-week-old male one to one. Embryos were collected from the oviduct of female mice with a vaginal plug in the morning, and granule cells were removed by digestion with 300 μg/mL hyaluronidase (Sigma，H4272) in M2 media (Merck Millipore, MR-015-D). Fertilized embryos with the second polar body were picked out and cultured in 30 μL KSOM (Merck Millipore, MR-121-D) droplet covered by mineral oil (Sigma, M8410) in 37°C incubator filled with 5% CO_2_.

Developmental stages of embryos were defined as follows: early 1-cell stage, 18-21 hours post HCG injection (hpi) or 9-12 hours post fertilization (hpf); late 1-cell stage, 26-29 hpi or 17-20 hpf; late 2-cell stage, 48-50 hpi or 39-41 hpf.

**Drug treatments**

To inhibit transcription, embryos with PB2 were treated in 100 μM α-amanitin (Cayman, 17898, dissolved in water) in KSOM for 3 h during late 1-cell stage or 2-cell stage. Embryos treated by α-amanitin survived through the first cell cleavage but could not develop to 4-cell or later stages. To disrupt microtubules, embryos with PB2 were treated in 25 μM nocodazole (Selleck, S2775, dissolved in dimethylsufoxide (DMSO)) in KSOM during early 1-cell stage; addition of DMSO was used as the control. After 3-h treatment, embryos were washed with normal KSOM three times.

**Nascent RNA and protein detection**

Cell-Light^TM^ EU Apollo567 RNA imaging kit (RIBOBIO, C10316-1) was used to label and detect nascent RNA in mouse embryos. Embryos at each stage were treated with 500 μM EU/KSOM for 3 h. After washing 3 times in KSOM, embryos were fixed with 4% paraformaldehyde and permeabilized with 0.5% Triton X-100 for 30 min at room temperature. After staining with EU staining solution under dark condition for 30 min, embryos were washed 3 times with PBS containing 0.5% Triton X-100.

To identify newly synthesized peptides in embryos, embryos were treated in 1 μg/mL puromycin (Merck Millipore, 540411-25MG) diluted with KSOM medium for 3 h at each stage before fixation in 4% paraformaldehyde. Then, incorporated puromycin was detected by immunostaining with anti-Puromycin Clone12D10 AF488 (Merck Millipore, MABE343- AF488, 1:200) antibody after cell permeabilization in 0.5% Triton X-100/PBS. Nuclei/DNA were stained with DAPI (Enzo, BML-AP402-0010) or Hoechst 33342 (Sigma, B2261). All the images were taken under a Zeiss 710 META confocal microscope and figures were processed by Imaris.

**Polar body injection**

The needle tip for polar body injection should be thinner than that for zygote injection. During injection, PB2 should lie on the 3 o’clock direction. Importantly, the embryo holder, PB2 and needle tip should be in a line at the same focal plan, so that the needle could easily penetrate into PB2. Besides, a gentle electric shock from Eppendorf PiezoXpert was needed. Thick needle tip, strong electric shock or large injection volume should be avoided as they would break PB2.

**Injection of protein and RNA**

Protein or RNA could be directly injected into PB2 or the zygote at the 1-cell stage. For tracing pbB or npbB descendants, *nls-mCherry* RNA was injected into pbB or npbB at the late 2-cell stage; if injected at the early 2-cell stage, the Nls-mCherry protein signal might transmit to the uninjected blastomere, losing labeling specificity.

*Cy5-miR-21-5p* (5’-UAGCUUAUCAGACUGAUGUUGA3-’) and *Cy5-miR21-3p* (5’-CAACAGCAGUCGAUGGGCUGUC-3’) were synthesized by GenScript. FITC-morpholino (5’-AATTCTAGATGTGACAAGCCAATGC-3’) was purchased from Gene Tools, LLC. This morpholino was designed as a control in an independent project and mouse zygotes injected with it developed normally during in vitro culture.

Concentrations of materials for injection were as follows: Nls-mCherry protein, 2 mg/ml; *nls-mCherry* mRNA and Cy5-labeled longer RNAs, 200 ng/μl; Cy5-*miR21*, 25 μM; morpholino, 1 μg/μl. The injection volume was approximately 4 pL per zygote/blastomere (with a droplet diameter of about 20 μm) or 0.1 pL per PB2 (with a droplet diameter of about 6 μm).

**pbB and npbB labeling by oil injection**

A drop of olive oil (Macklin, O815210) with a size slightly smaller than PB2 was injected into 1-cell embryos after 23 hpi. Earlier injection likely causes contact of the oil droplet to the pronucleus, in which case the oil droplet would migrate together with pronucleus and the embryo could not survive through the first cleavage. The needle for oil injection (World Precision Instruments, TW100F-4) should be thinner than that for RNA injection so that the size of an oil droplet could be easily controlled. Injection pressure from Eppendorf FemtoJet 4i ranged from 2000 to 3000 hPa and holding pressure ranged from 700 to 800 hPa depending on the opening width of the needle. No matter oil was injected into the zygote near PB2 (PB side) or opposite to it (NPB side), the position of the oil droplet should be on the line of the animal-vegetal axis and should never drift away from the axis. After completion of all injections, the position of the oil droplet should be double-checked and embryos with wrong oil position should be discarded. At the 2-cell stage, PB side injection let the oil droplet sit in pbB and NPB side injection let it stay in npbB. The injected oil droplet was approximately 12 μm in diameter (about 1 pL).

**Cell fate analysis of preimplantation embryos**

The first approach was based on confocal microscopic observation of immunostained blastocysts. When oil-injected zygotes developed to the 2-cell stage, one blastomere was injected with *mCherry* mRNA. At the 4-cell stage, embryos with mCherry fluorescence in only two of cells were cultured further and fixed at E4.5 with 4% paraformaldehyde, while embryos with all cells showing mCherry fluorescence, which most likely resulted from flow of injected *mCherry* mRNA into the uninjected blastomere at the 2-cell stage, were discarded. The fixed blastocysts were immunostained by anti-Cdx2 (BioGenex, MU392A-UC, 1:200) and anti-mCherry (Easybio, BE2027, 1:200) antibodies with nuclei stained by Hoechst 33342. Then, blastocysts were observed by confocal microscopy and multiple Z-slices were acquired. By observing acquired images from different angles, Cdx2^+^;Hoechst 33342^+^ cells, which were located in the outer layer, were defined as TE cells; and Cdx2^-^;Hoechst 33342^+^, which were located in the inner part, were regarded as ICM cells. Number of mCherry^+^ and mCherry^-^ cells in the whole embryos and ICM cells were counted. Difference in cell proportion between pbB and npbB descendants in blastocyst or ICM was analyzed by paired-sample two-tail *t*-test in Excel.

The second approach was based on live imaging. 100 ng/μl *CAAX-gfp* mRNA was injected at the 1-cell stage to label the plasma membrane, followed by oil injection into the NPB side of the zygote. Then at the 2-cell stage, *nls-mCherry* mRNA was injected into pbB to label its descendants. Olympus FV3000 confocal microscope was used to take live imaging of the embryos from the 8-cell stage onward. Embryos were incubated inside the transparent incubation box with the upper temperature of 40°C and the stage temperature of 37°C, filled with 5% CO_2_. Photo series were taken at the interval of 15 min and the movies were processed by Imaris.

**Embryo splitting and reaggregation**

ZP of 2-cell stage embryos was removed by treatment with acid Tyrode’s solution (Merck Millipore, MR-004-D). Then, the embryos were immediately transferred to 37°C pre-heated 0.25% Trypsin (AMRESCO, 0458), and pbB and oil-labeled npbB were separated by gentle pipetting and washed in KSOM media twice. Two dissociated pbBs or npbBs, or one dissociated pbB and npbB were reaggregated in a gelatin-coated 96-well round-bottom plate (Corning, 3799) filled with 50 μl KSOM media. A single reaggregated embryo was cultured in one well to avoid embryo-embryo attachment.

**Polar body removal**

PB2 removal at the 1-cell stage was performed between 24-27 hpi and 2-cell stage PB2 removal was performed between 42-45 hpi. PB2 should not be removed during early 1-cell stage because this manipulation caused a leakage of the cytosol inside the zygote and damaged the embryo. For PB2 removal, the first step was to create a cleft in the ZP. We used a thin needle to penetrate the ZP near PB2, rubbing it on the surface of the embryo holder. After the embryo dropped from the thin needle, a cleft could be observed in the ZP. Next, a thick needle, whose diameter was smaller than PB2, was put into the embryo through the ZP cleft, PB2 was then sucked into the needle and the needle was pulled out gently. For control group, only a cleft was created in the ZP. Because of the cleft, embryos hatched earlier from ZP than untreated normal embryos, resulting in 8-shaped blastocysts.

**PB lysate preparation**

At the late 1-cell stage, ZP was removed by treating in acid Tyrode’s solution (Merck Millipore, MR-004-D). Then, the embryos were transferred to the medium containing 0.25% Trypsin (AMRESCO, 0458) immediately. After continuous pipetting in trypsin, PB2 was split from the embryo. 100 PB2s were collected and lysed together in 1 μL iced hypotonic extraction buffer containing 20 mM HEPES (pH 7.5), 1 mM magnesium acetate, 10 mM potassium acetate, 4 mM dithiothreitol, and EDTA-free Protease Inhibitor Cocktail (Roche, 04693132001)^1^. The supernatant was transferred to another RNase-free tube after centrifugation at 10,000g for 10 min at 4°C.

**in vitro postimplantation embryo culture**

In vitro culture of the mouse blastocyst followed the protocol published by Ma et al. (2019)^2^, which was optimized from that reported by Bedzhov et al. in 2014^3^. The zygotes in each group were first cultured in vitro to form E4.5 blastocysts, which were then cultured on Matrigel (BioCoat, 356230)-coated dishes, filled with CMRL medium (Invitrogen, 11530-037) containing sodium pyruvate (Gibco, 11360070), GlutaMAX (Gibco, 35050079), NEAA (Gibco, 11140050), B-27 (Invitrogen, 17504-044), N-2 (Invitrogen, 17502-048), penicillin and streptomycin. During the first 2 days of culture, 10% fetal bovine serum (FBS, Merck Millipore, ES-009-B) was supplied to the medium. At the third day of culture, concentration of FBS was changed to 20%. At the fourth day, FBS was changed to 30% KSR (Thermo Fisher, 10828028) to support embryonic development.

**Embryo transplantation**

Blastulas at 4-8 cell stages (prior to compaction) were transplanted to a single oviduct of a pseudo-pregnant female mouse (ICR) on the day of vaginal plug appearance. Before surgery, surrogate mice were anesthetized with 300 mg/kg Avertin (Sigma, T48402). Each surrogate mouse received 12 to 17 embryos. 9 days after transplantation, the uterus was dissected to find and collect embryos.

**RNA sequencing and data processing**

70 PB2s and zygotes of 1-cell mouse embryos were collected as described in PB lysate preparation. Samples were collected into lysis buffer from Vazyme Single Cell Full Length mRNA-Amplification Kit (N712) for mRNA amplification. RNA libraries were prepared for sequencing using the TruePrep^TM^ DNA Library Prep Kit V2 for Illumina (Vazyme Biotech Co.,Ltd, TD503) protocol. The libraries were quantified by Qubit 2.0 and Agilent 2100, and sequenced in the Illumina NovaSeq 6000 system.

After sequencing, the Illumina Casava software was used for base-calling. RNA-Seq reads were aligned to the reference genome OryCun2.0 using HISAT2 (v2.0.4) with default parameters. Gene-level read counts were calculated using HTSeq (v0.6.1p1) based on the Ensembl gene annotation v85. DEseq2 (v1.18.1) was used for data normalization to identify differentially expressed genes.

**RT-qPCR analysis**

An aliquot of the PB2 and zygote cDNAs, which were amplified using the Vazyme Signle Cell Full Length mRNA-Amplification Kit (N712) for RNA sequencing, was used as template. A set of pluripotency-related genes that were significantly up-regulated in PB2 as revealed by RNA sequencing data were examined using specific primers (Table S1) and *Actb* as an internal control. Real-time PCR was performed using qPCR master mix with ROX (Biotium, 31042). The relative expression level was calculated by the 2^-∆∆Ct^ method.

**Immunostaining and antibodies**

For immunostaining of α-Tubulin, embryos were fixed in methyl alcohol at -20°C overnight. Embryos for immunostaining with the other antibodies were fixed in 4% paraformaldehyde at 4°C overnight. After fixation, embryos were permeabilized in PBS containing 0.5% Triton X-100 for 30 min at room. The following antibodies were used: anti-RNA polymerase II (Abcam, Ab817, 1:200); anti-RNA pol II CTD phospho Ser5 (Active motif, am61085, 1:500); anti-ribosomal RNA (DSHB, 1:24); anti-α-Tubulin (Sigma, T6199, 1:1000); anti-Cdx2 (BioGenex, MU392A-UC, 1:200); anti-Sox2 (Abcam, ab92494, 1:100); anti-active Yap1 (Abcam, ab205270, 1:500); anti-mCherry (Easybio, BE2026 and BE2027, 1:200); anti-Puromycin Clone12D10 AF488(Merck Millipore,MABE343-AF488, 1:200); anti-Mouse IgG(H+L) AF488 (Jackson, 115-545-003, 1:200); anti-Rabbit IgG(H+L) AF647 (Jackson, 111-605-003, 1:200). All the images were taken under a Zeiss 710 META confocal microscope and figures were processed by Imaris.

**Statistical analysis**

Chi-square test was applied to analyze the difference in the rate of in vitro cultured embryos or in vivo implanted embryos. All the other analyses were done by two-tail *t*-test in Excel. Particularly, proportions of pbB and npbB descendants in blastocyst and ICM were analyzed as paired-samples.

1 Zeenko, V. V. *et al.* An efficient in vitro translation system from mammalian cells lacking the translational inhibition caused by eIF2 phosphorylation. *RNA* **14**, 593-602, doi:10.1261/rna.825008 (2008).

2 Ma, H. *et al.* In vitro culture of cynomolgus monkey embryos beyond early gastrulation. *Science* **366**, doi:10.1126/science.aax7890 (2019).

3 Bedzhov, I., Leung, C. Y., Bialecka, M. & Zernicka-Goetz, M. In vitro culture of mouse blastocysts beyond the implantation stages. *Nat Protoc* **9**, 2732-2739, doi:10.1038/nprot.2014.186 (2014).

# Table S1. Primers for RT-PCR

| **Gene**  **name** | **Forward primer** | **Reverse primer** |
| --- | --- | --- |
| Actb | AGATCAAGATCATTGCTCCTC | TGTAAAACGCAGCTCAGTAA |
| Wnt7a | GTGGACTGCTTGAGGTCCTGG | GGATAGGAGCACGTCCTGGAGA |
| Dvl2 | GGACTGGCTGTACCATCACG | GCTGGATACTGGTAGGAGAAGGTG |
| Wnt10b | GCGTCTTCTCTACCTACAGCTCC | GTCTGAGGCTTCCCTGCCC |
| Ctnnb1 | CACAGCTCCCCTGACAGAG | GGTCCATCCCCAAGGCATC |
| Tcf3 | GTGCATCAGGACGGTTCTGG | GGCACGATGTGCTGGTGG |
| Axin | CTCTCCTGACAGTGGGCATG | CCTGCGTTCTCGGAATAGCTC |
| Smad3 | CCAAGTAATCGCGCATCAACG | CACATAGCCATCCACAGTCACAAC |
| Smad1 | AGATGCCAGCTGACACACC | TTCCGGTTAACGTTGGAGAGC |
| Acvr2b | CAGGAGGTGGTTGTCCACAAGAAG | AGATCCACTGAGTCTGGAGAGACG |
| Smad4 | CGGAGTGCCTCAGTGACAG | AGGTGTAGCTCAATCCAGCAG |
| Map2k2 | GGCAGGGGACCTATGCCC | CATGCAGGGCACTGCCTTTG |
| Raf1 | GACTACATCCCCAAGGCTACCAG | CCTCTGGAGCTGAGCAGGC |
| Mapk3 | CCCTGGAAGCCATGAGAGATG | ACATACTCCGTCAGAAAGCCAG |
| Stat3 | GGAATGAAGGGTACATCATGGG | TGGTCGCATCCATGATCTTATAGC |
| Akt1 | GAGTGTGTGGACAGTGAGCG | CTGACCTGGCTGGGAGGTAG |
| Otx1 | GGTCAAGAAGAAGTCGTCTCCAG | GTGGCTCTGGCACCGATAC |
| Onecut1 | GGCAGTTCATCGTCCTCATCGAG | GCCCCGCTGAAGTATGTGTC |
| Pou5f1 | CACTTCACCACACTCTACTCAGTCC | GCCAGGCTCCTGATCAACAG |


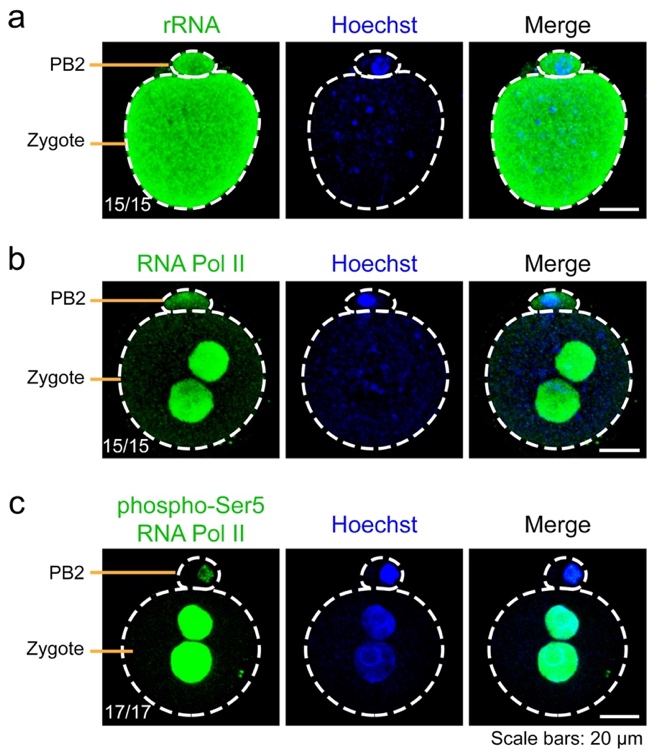


**Fig. S1. Existence of rRNA and RNA Polymerase II in PB2.** (a**)** Immunostaining result of rRNA with rRNA antibody. (b and c)**,** Immunostaining results of RNA Polymerase II with an anti-RNA Pol II CTD repeat YSPTSPS antibody (b) or anti-RNA Pol II CTD phospho Ser5 (active form) antibody (c). Immunostained embryos were observed by confocal microscopy. The ratio of embryos with the representative pattern was indicated.


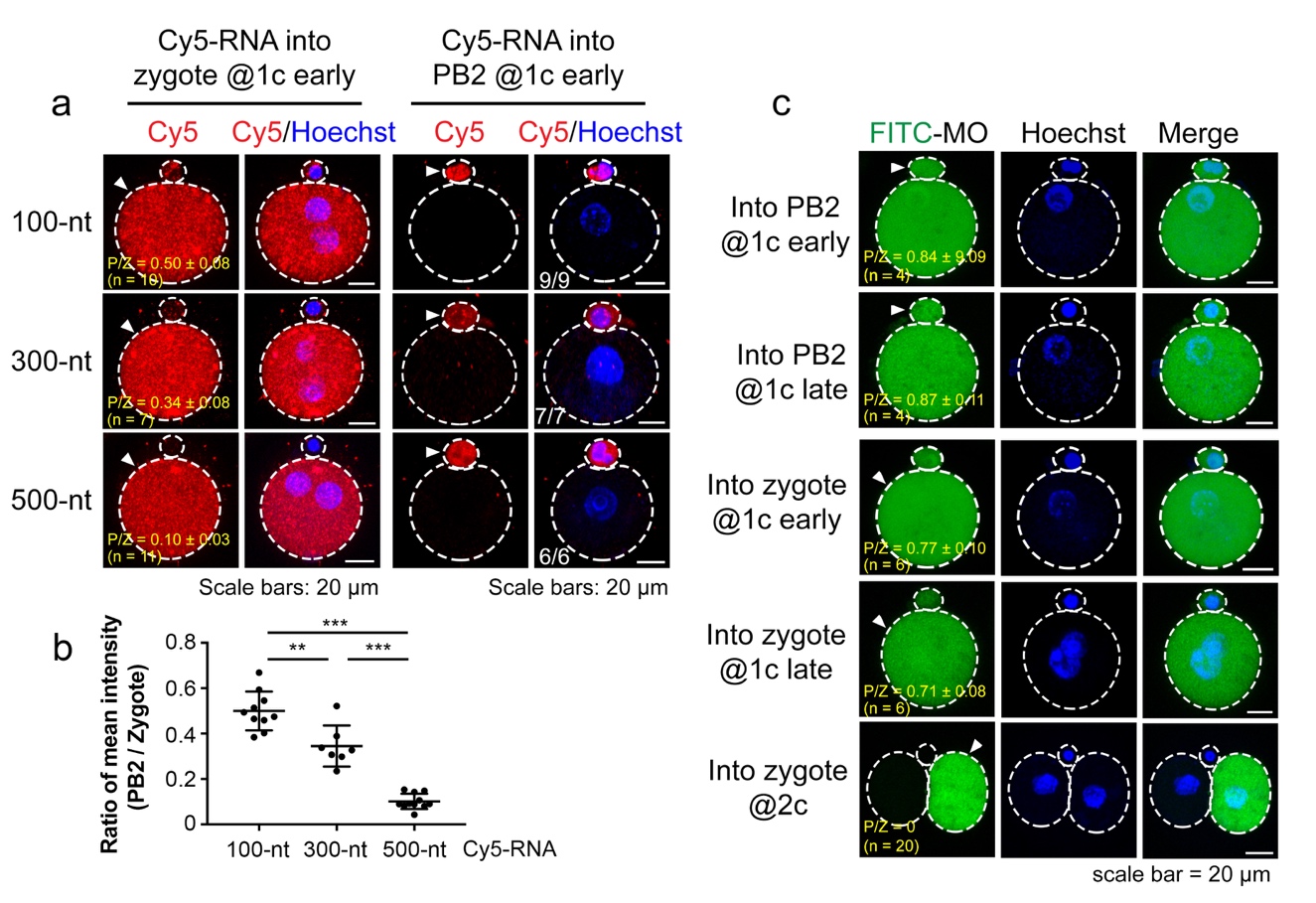


**Fig. S2. Transport of RNAs with different lengths between PB2 and the zygote.** (a and b) Transport of 100-nt, 300-nt or 500-nt RNA. RNAs derived from *mCherry* sequence were labeled with Cy5 and injected into the zygote (left panel) or PB2 (right) at the early 1-cell stage as indicated by arrowheads (a). P/Z, ratio of mean signal intensity in PB2 to that in the zygote. The P/Z ratios among different RNAs were compared statistically (b). **, p < 0.01; ***, p < 0.001. (c) Transport of a 25-nt morpholino-modified oligonucleotide conjugated with FITC (FITC-MO). FITC-MO was injected into the zygote or PB2 at early or late 1-cell stage or into one of blastomeres at the 2-cell stage as indicated by arrowheads. Embryos were observed by confocal microscopy around 4 h post-injection. The ratio of embryos with the representative pattern was indicated.


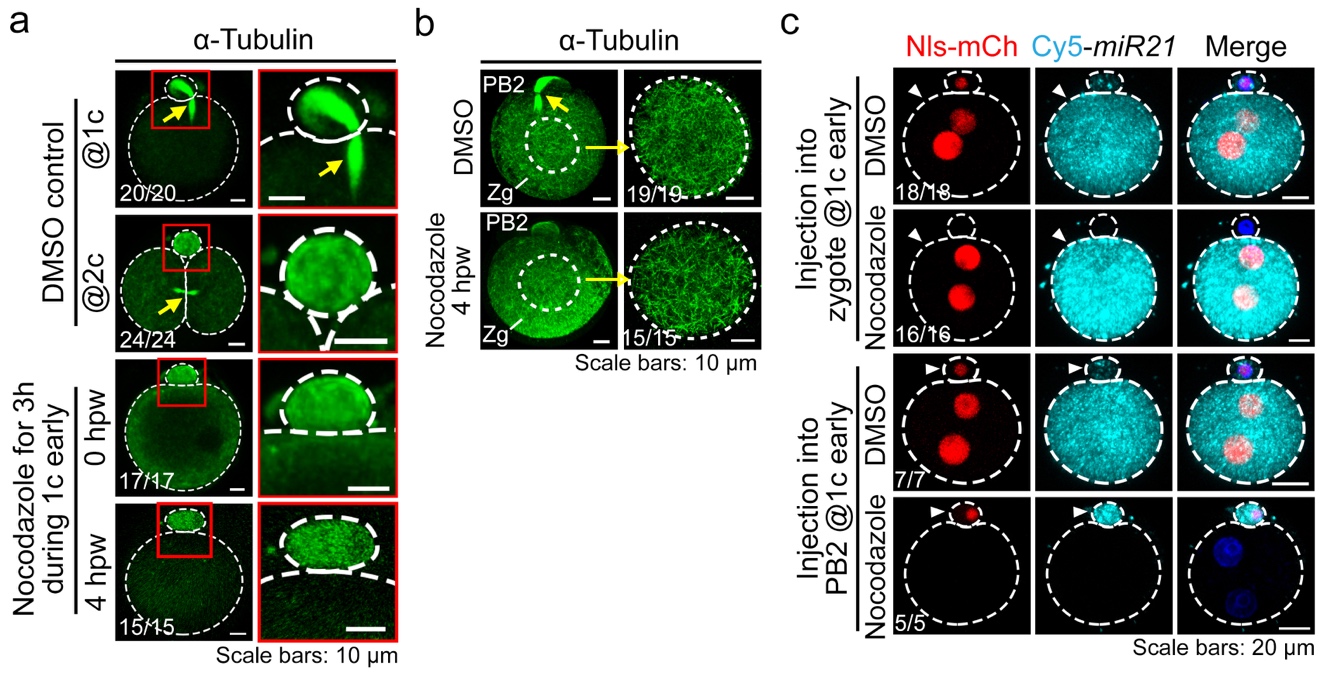


**Fig. S3. Blockage of material exchange between PB2 and the zygote by transient nocodazole treatment might be due to Disruption of the midbody.** (a and b) Disruption of the midbody by nocodazole treatment. Embryos were incubated in the presence of nocodazole or DMSO (control) for 3 h during the first cleavage period, then washed and further incubated in normal medium for 4 h until fixed for immunostaining with α-tubulin antibody. The right columns showed the enlarged area boxed in the left columns. The midbody was indicated by an arrow. hpw, hours post-wash. The immunostained embryos were observed by confocal microscopy using a 20x len (a) or 100x oil len (b). Note that the midbody was still absent at 4 h postwash (hpw) while re- formed microtubules in the cytoplasm of the zygote (b). (c) Inhibition of protein and microRNA transport between PB2 and the zygote by nocodazole treatment. Embryos at the early 1-cell stage were incubated in the presence of nocodazole or DMSO (control) for 3 h and then injected with Nls-mCherry protein and Cy5-*miR21-5p* RNA into the zygote or PB2 as indicated by arrowheads. Approximately 4 h post-injection, the embryos were observed by confocal microscopy. The ratio of observed embryos with the representative pattern were indicated in the bottom left corner in the first column.


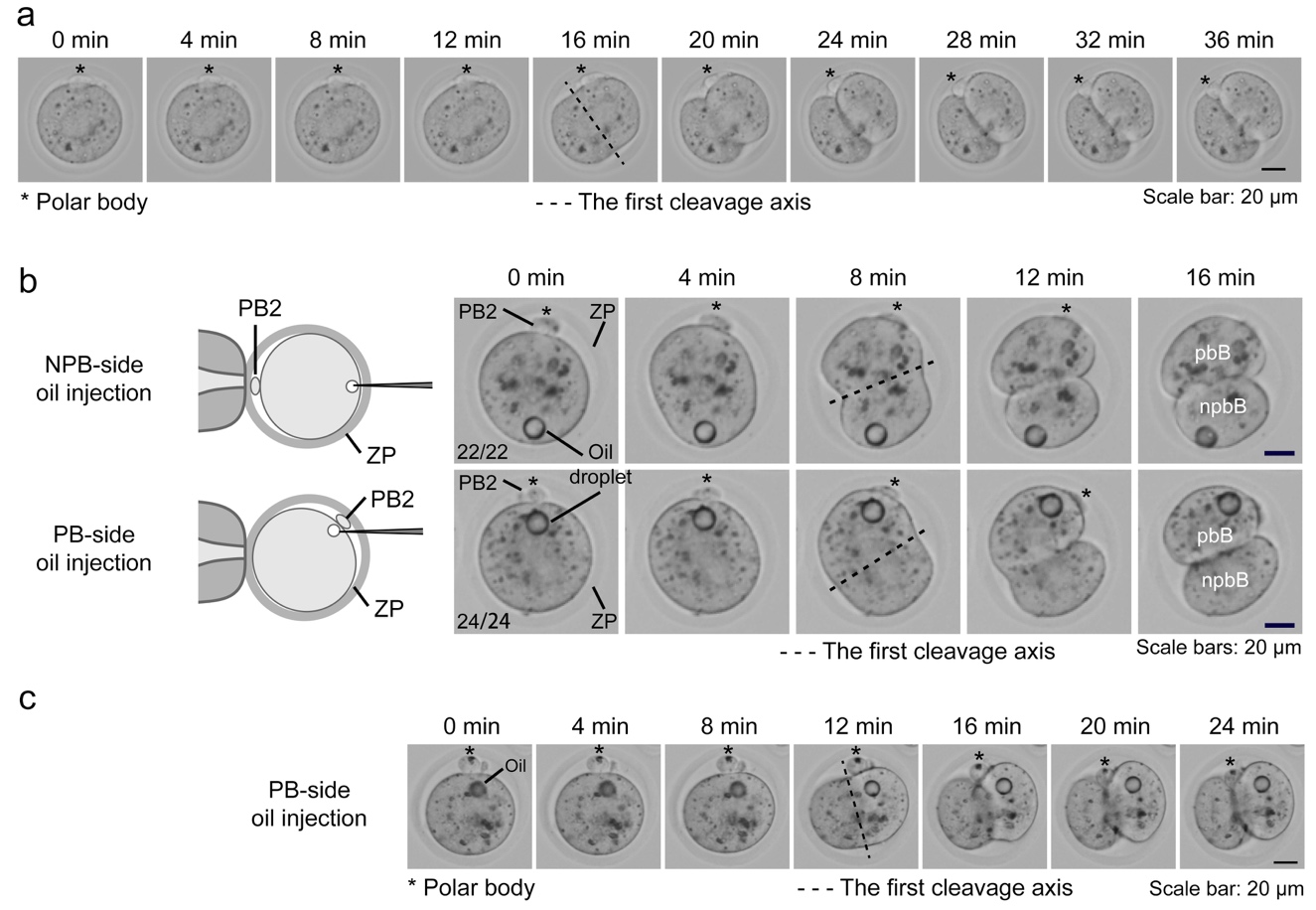


**Fig. S4. Injected olive oil droplet remains stationary during embryonic development.** (a) An example of an unlabeled zygote with PB2 located between two blastomeres at the 2-cell stage. In this case, it is not possible to determine at the 2-cell stage which blastomere is derived from the zygote part connected to PB2, making labeling of one blastomere meaningless for studying asymmetric fates of two blastomeres. (b) Oil droplet helps determine PB2-connected or unconnected area within the zygote. Left panel, illustrations of two different oil injection positions. Right top panel, an oil droplet injected into the NPB side (opposite to PB2) of the zygote; right bottom panel, an oil droplet injected into the PB2 side (just below PB2) of the zygote. Note that PB2 (indicated by *) moved away from its original position while the oil droplet stayed in the original position during development. See also Video S2. (c) An example of a PB-side labeled zygote with PB2 located between two blastomeres at the 2-cell stage. Note that the first cleavage furrow forms adjacent to PB2, and PB2 subsequently move to the junction site between two blastomeres. All embryos were shown as live images.


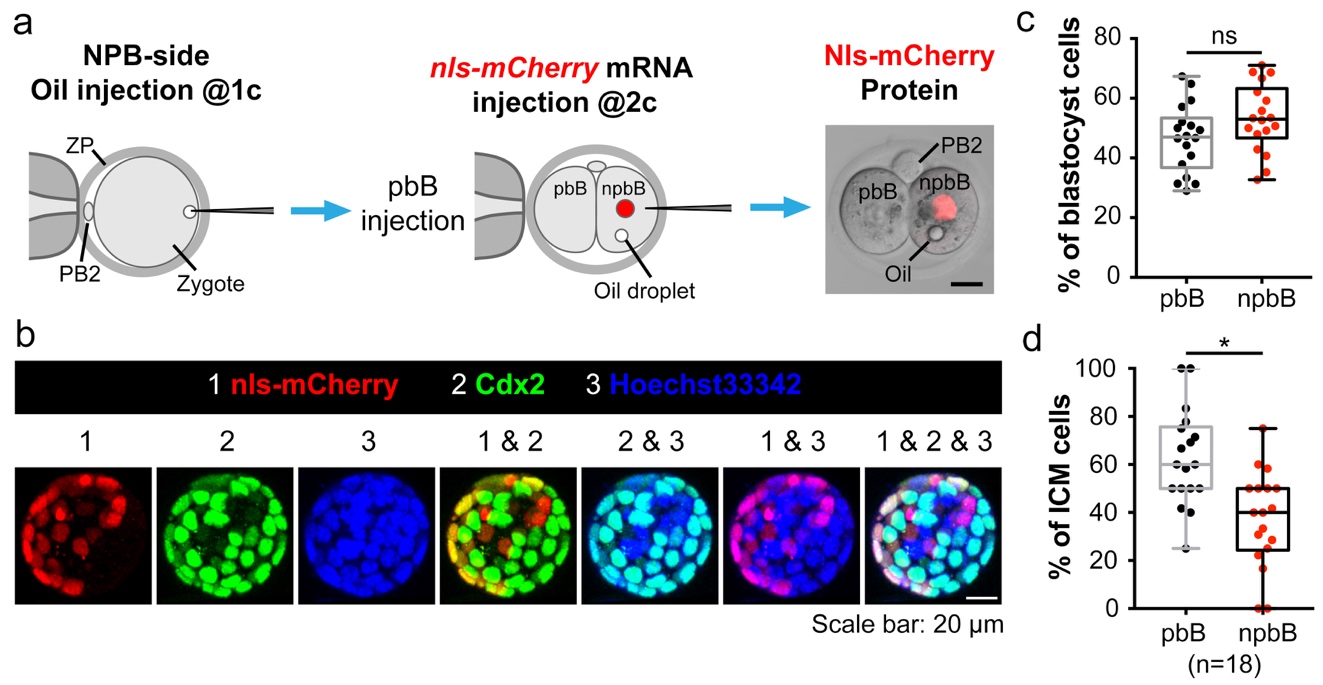


**Fig. S5. pbB without oil and RNA injection still contributes more descendants to the ICM in blastocysts than npbB.** (a) Illustration of injection procedure. Injections were performed in a way so that both the oil droplet and *nls-mCherry* mRNA were placed into npbB at the 2-cell stage to avoid possible interference of injection and injected materials with the fate of pbB. An injected embryo was shown on the right as an example. (b) Example of immunostained blastocysts. The injected embryos at blastocyst stage were subjected to immunostaining with mCherry and Cdx2 antibodies. DNA/nucleus was stained with Hoechst 33342. ICM (inner) cells were Hoechst- positive and Cdx2-negative. (c and d) Percentage of pbB- or npbB-derived blastocyst cells (**c**) or ICM cells (d). Each dot represented the percentage from one blastocyst. Box and whiskers plot, from min to max with the median indicated by the center line; box limits, upper and lower quartiles; n, number of observed blastocysts. Statistical significance: ns, nonsignificant (P > 0.05); *, P < 0.05.


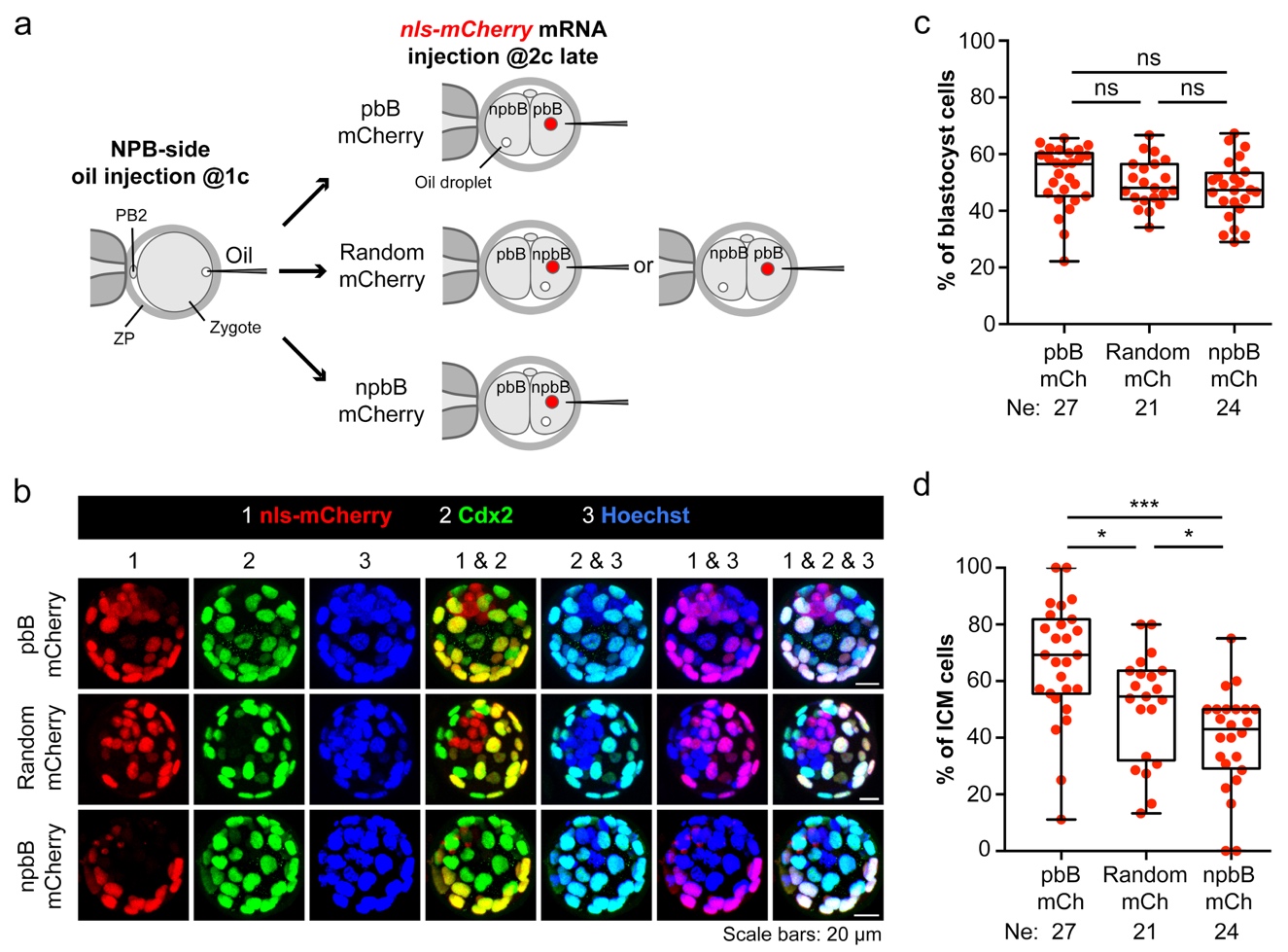


**Fig. S6. Random labeling of two blastomeres at the 2-cell stage is unable to detect biased fate differentiation at blastocyst stages.** (a) Illustration of injection procedures. After labeling NPB side by oil injection at the 1-cell stage, pbB (upper panel) or npbB (lower panel) was injected with *nls-mCherry* mRNA, or *nls-mCherry* mRNA was randomly injected to pbB or npbB (middle panel). (b) Examples of immunostained blastocysts in each group with Cdx2 and mCherry antibodies. (c and d) Percentage of blastocyst cells (c) or ICM cells (d) originated from pbB or npbB or random blastomere. Each dot represented the percentage from one blastocyst. Statistical significance: ns, nonsignificant (P > 0.05); *, P < 0.05; ***, P < 0.001. Note that random labeling failed to reveal biased contribution of labeled blastomere’s descendants to the ICM.


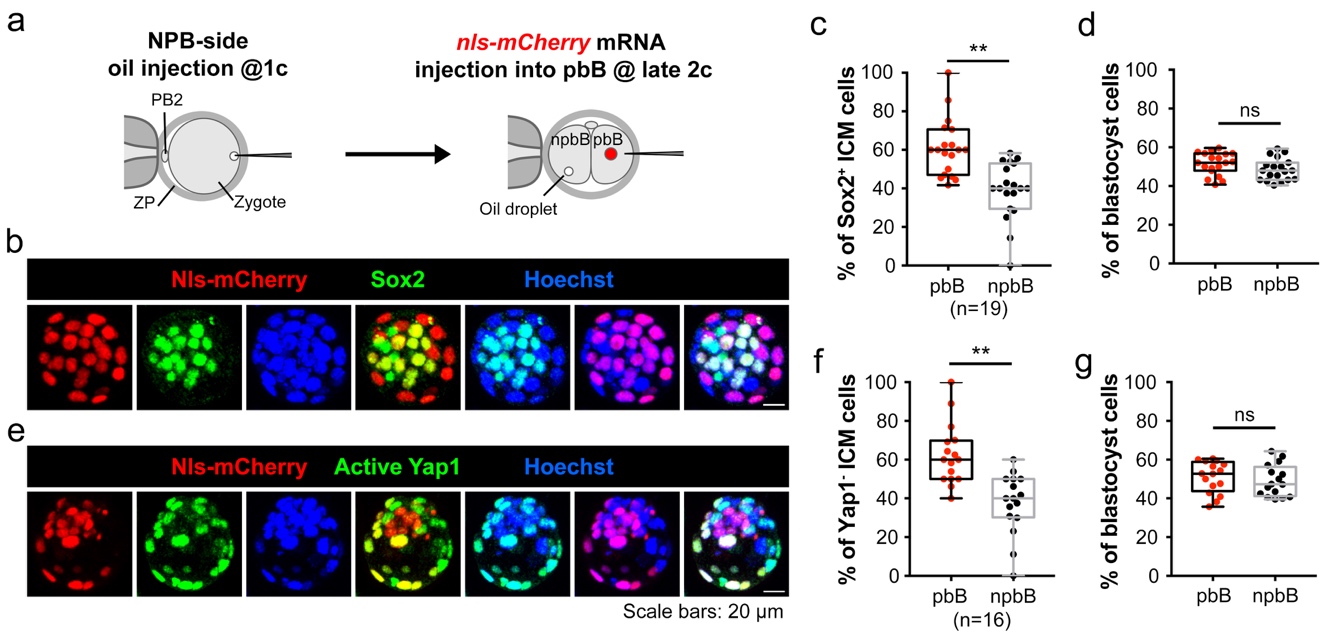


**Fig. S7. Detection of endogenous Sox2 and active Yap1 also reveals fate bias of pbB and npbB.** (a) Illustration of injection procedure. (b and e) The blastocysts were immunostained with mCherry and Sox2 (an ICM marker) antibodies (b) or mCherry and active Yap1 antibodies (e). (c and d) Percentage of Sox2^+^-ICM cells (c) or percentage of pbB- or npbB-derived blastocyst cells (d) in Sox2-immunostained blastocysts. (f and g) Percentage of Sox2^+^-ICM cells (f) or percentage of pbB- or npbB- derived blastocyst cells (g) in active Yap1-immunostained blastocysts. n, the number of observed blastocysts. Statistical significance: ns, nonsignificant (P > 0.05); **, P < 0.01.


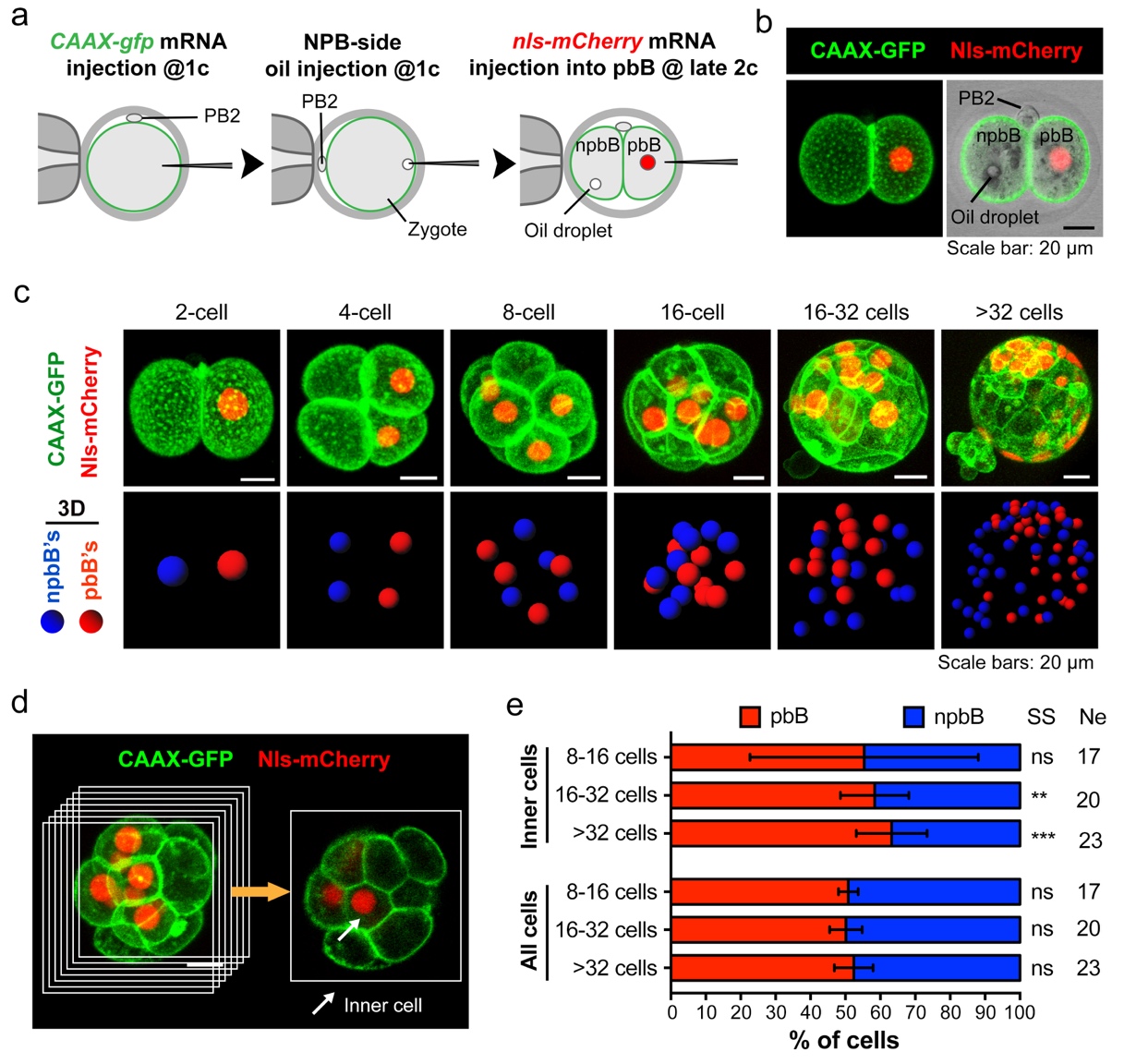


**Fig. S8. Developmental fate recognition of pbB’s and npbB’s descendants by confocal microscopic live imaging.** (a) Illustration of injection procedure. *Caax-gfp* mRNA was injected into the zygote at the 1-cell stage, followed by oil injection into the NPB side. Then at late 2-cell stage, pbB was labeled by injecting with *nls-mCherry* mRNA. (b) CAAX-GFP, Nls-mCherry and oil droplet locations in a live embryo at the 2-cell stage. (c) Confocal microscopic time-lapse images and 3-dimension (3D) reconstitutes at indicated stages (see also Video S4). Upper panel, confocal z-stack live images with Nls-mCherry signal in nuclei of pbB-derived cells; lower panel, 3D- reconstitutes of nuclei with pbB colored by red for Nls-mCherry and npbB colored by blue for no signal. (d) An example of live embryo observation. Left panel, many confocal Z-slices were obtained to constitute one Z-stack image of a 16-cell stage embryo; left panel, one slice to show clear location of an inner cell (indicated by an arrow). (e) Percentage of pbB and npbB in inner cells (upper panel) or all cells (lower panel) in each cleavage period. The judgement of inner or outer cells was done as illustrated in (d). SS, statistical significance. ns, nonsignificant (P > 0.05); **, P < 0.01;

***, P < 0.001. Ne, number of observed embryos. Note that, to avoid damaging effect of repetitive laser irradiation during a long imaging period, one batch of embryos was divided into three groups, each for imaging in one period (8- to 16-cell, 16- to 32-cell, or 32- to 64-cell).


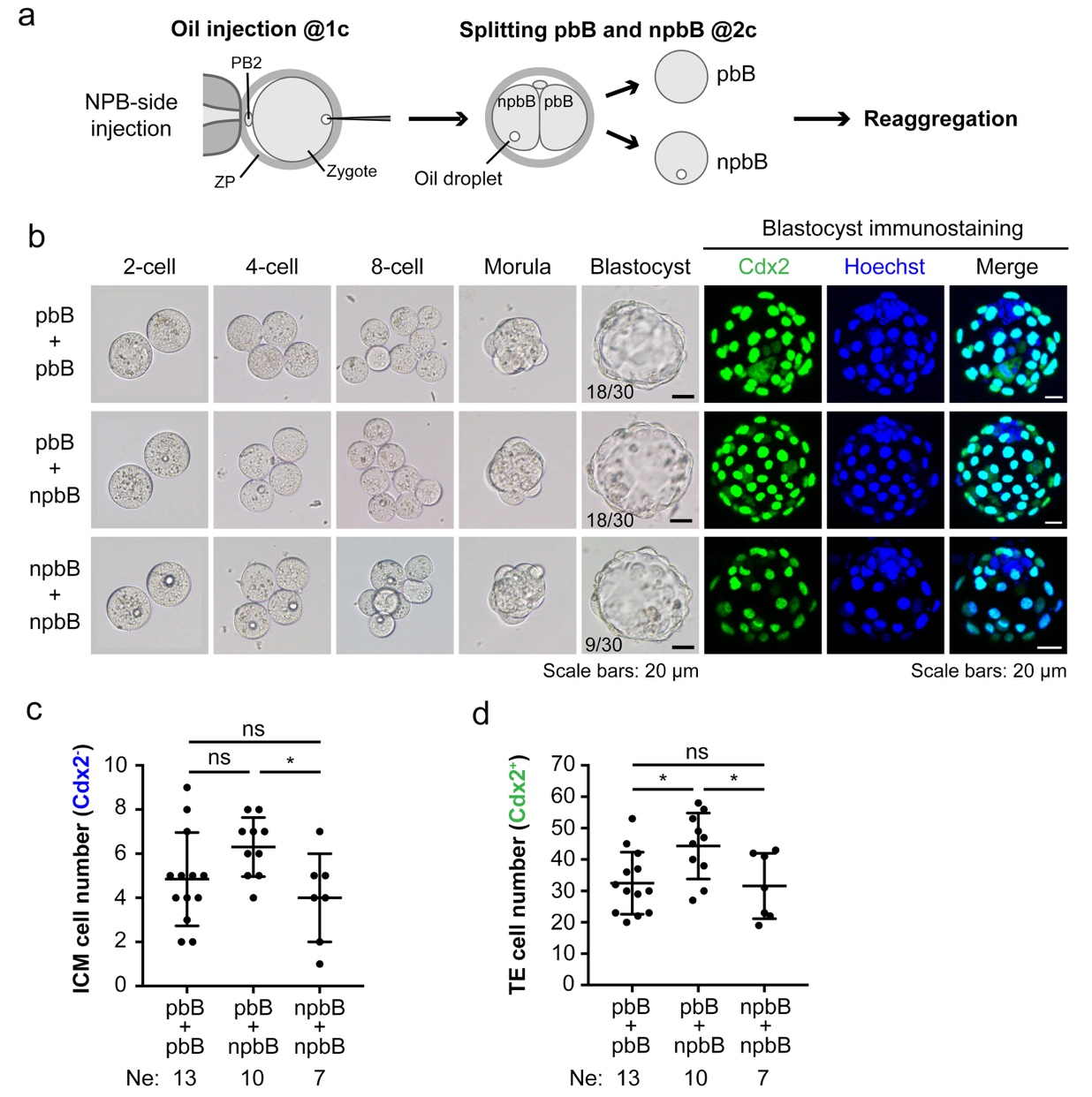


**Fig. S9. Developmental potential test of reaggregates between pbB and npbB.** (a) Illustration of experimental procedure. (b) Morphology of reaggregates at different stages and Cdx2-immunostained blastocysts. A group of about 10 2-cell stage embryos were dissociated individually and two dissociated blastomeres were transferred to a U- shaped well of a 96-well dish for culture until blastocyst stages. pbB + pbB, reaggregate from two dissociated pbBs, pbB + npbB, reaggregate from one dissociated pbB and one dissociated npbB; npbB + npbB, reaggregate from two dissociated npbBs. The ratio of survival blastocysts was indicated. The survival blastocysts were immunostained with Cdx2 antibody and nuclei were stained with Hoechst, followed by confocal microscopic observation with multiple Z-slices. (c and d) ICM (c) and TE (d) cell numbers of reaggregate-derived blastocysts. ns, nonsignificant (P > 0.05); *, P < 0.05.


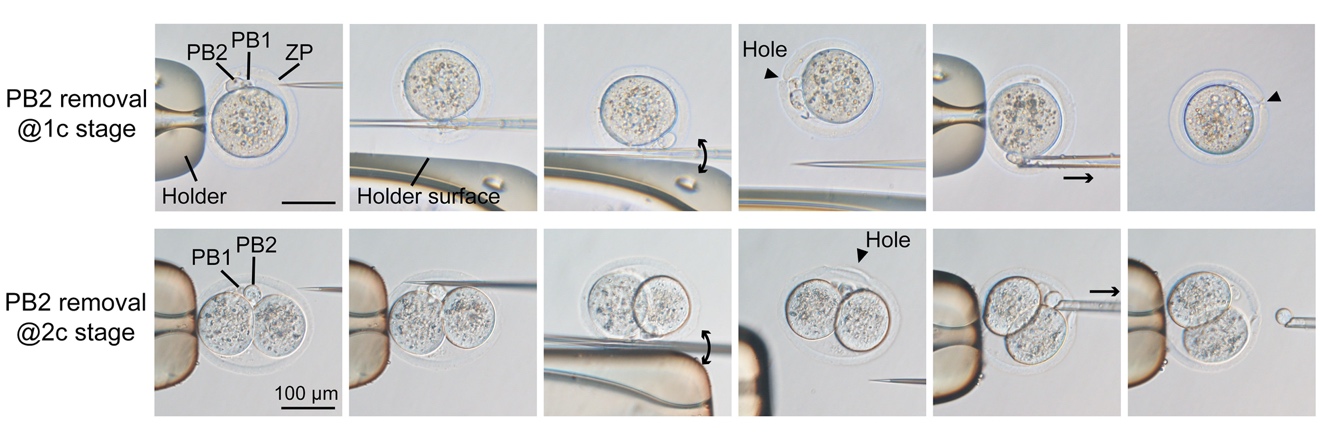


**Fig. S10. PB2 Removal procedure.** Top panel, PB2 removal at the 1-cell stage; bottom panel, PB2 removal at the 2-cell stage. A sharp needle was penetrated into zona pellucida (ZP); a hole was made by repetitive rubbing ZP against the holder surface; PB2 was sucked using an injection needle with a wide opening. See also Video S5.


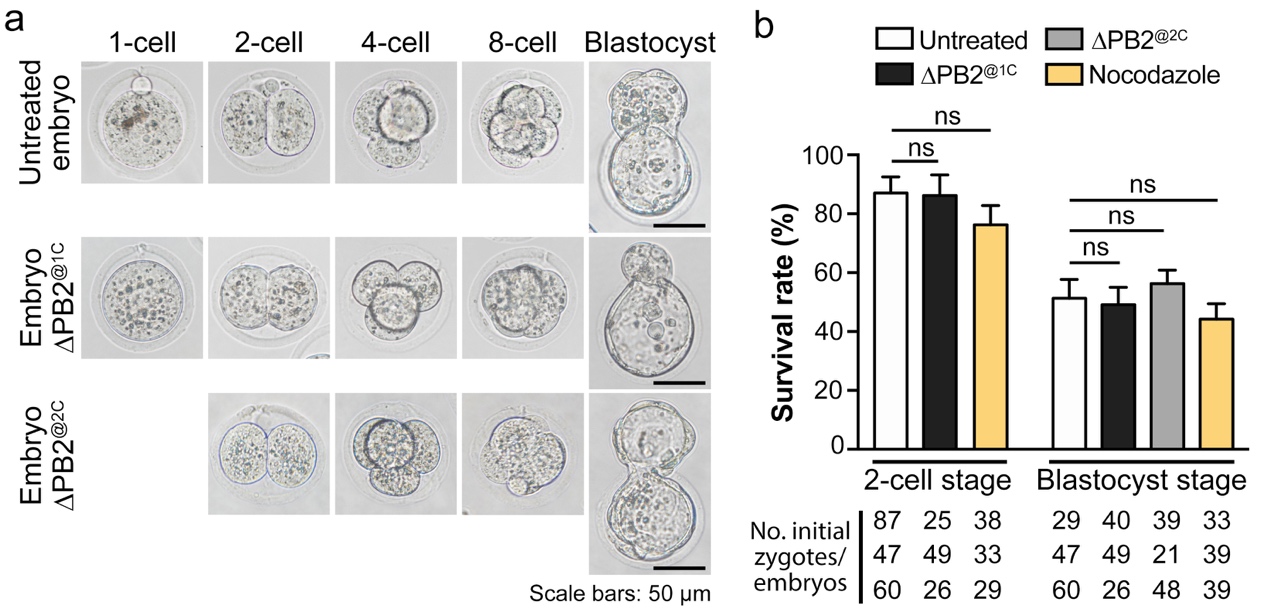


**Fig. S11. PB2 removal or nocodazole treatment does not affect preimplantation embryonic development.** (a) Morphology of representative embryos at different preimplantation stages. Untreated embryo and embryos with PB2 removed at 1-cell or 2-cell stage were shown. Embryos that were treated with nocodazole for 3 h during the first cleavage looked like untreated embryos and were not shown here. (b) Survival rates of embryos in different groups at the 2-cell and blastocyst stages. The survival rate of each group was averaged from three independent experiments with initial zygote/embryo numbers indicated. ns, not statistically significant (P > 0.05).


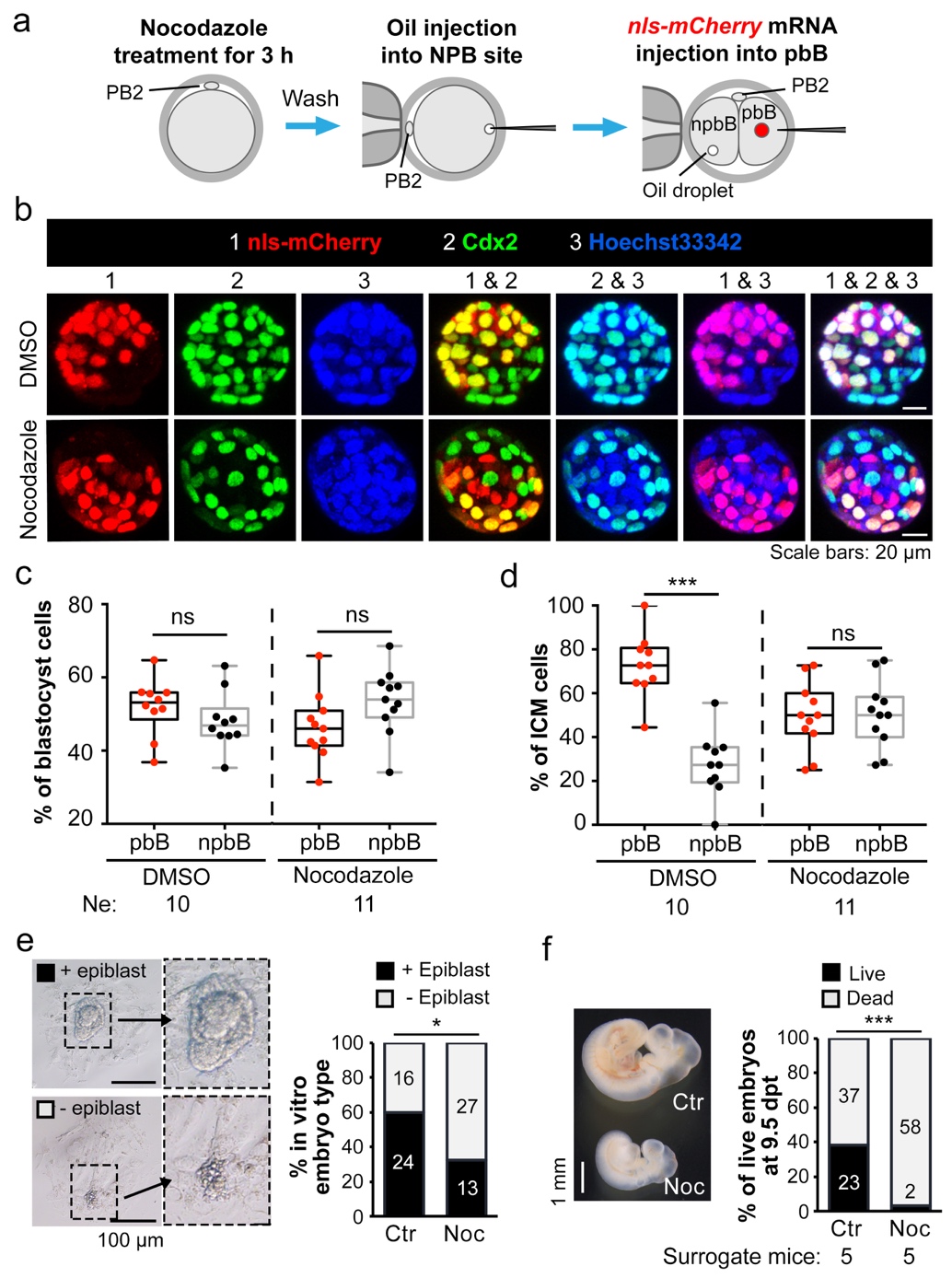


**Fig. S12. Transient nocodazole treatment affects preimplantation cell fate determination and postimplantation development.** (a) Illustration of nocodazole treatment and injection procedure. (b) Examples of immunostained blastocysts. (c and d) Box and whiskers plots showing percentage of pbB- or npbB-derived blastocyst cells

(**c**) or ICM cells (d). Each dot represented the percentage from one blastocyst. (e) Nocodazole (Noc) treatment causes abnormal in vitro development of embryos. Left, examples of embryos with (+) or without (-) epiblast-like structure after in vitro culture until 7.5 days post-fertilization. Right, ratio of two types of cultured embryos. (f) Nocodazole treatment impaired postimplantation embryonic development. Nocodazole-treated (Noc) or DMSO-treated (Ctr) embryos were transferred to surrogate mice, and percentage of live embryos with normal tissues at 9.5 days post- transplantation was calculated. Representative embryos were shown in the left panel. The dead embryos included non-implanted blastulas as well as implanted but deformed embryos. In bar graphs, total embryo numbers (pooled from several batches) were shown within bar areas. Statistical significance: ns, nonsignificant (P > 0.05); *, P <0.05; ***, P < 0.001.


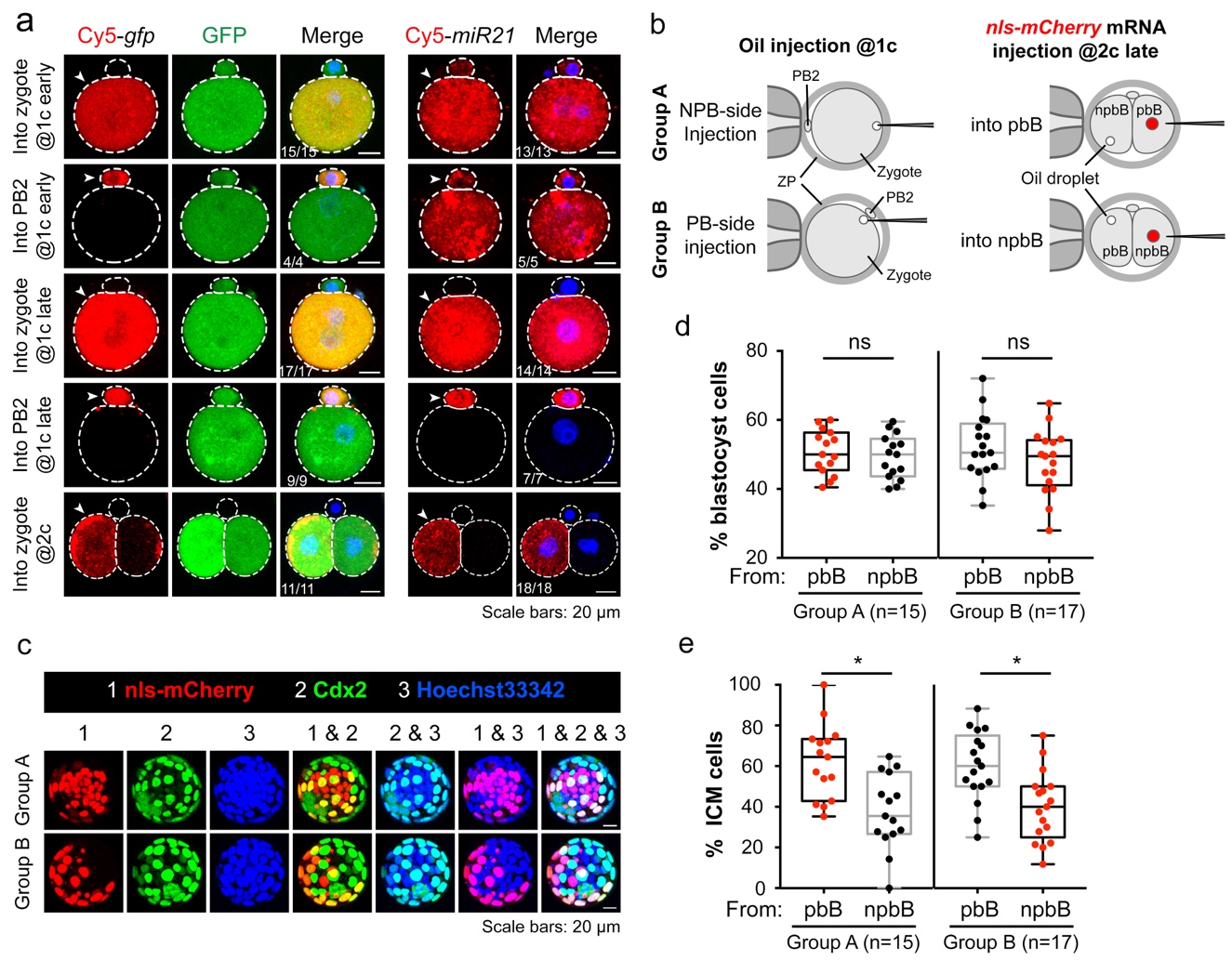


**Fig. S13. PB2 in the zygote of the ICR strain plays a similar role in preimplantation cell fate determination.** (a) Protein and microRNA transport between PB2 and the zygote. Injection of Cy5-labeled *gfp* mRNA (left panel) and *microRNA-21* (right panel) into PB2 or the zygote at different stages. All images were taken with confocal microscopy 3 hours post-injection. Nuclei were stained with Hoechst33342. The ratio of embryos with the representative pattern was indicated. (b) Illustration of injection strategies. **c,** Examples of immunostained blastocysts. (d and e) Percentage of pbB- or npbB-derived blastocyst cells (d) or ICM cells (e). Each dot represented the percentage from one blastocyst. Statistical significance: ns, nonsignificant (P > 0.05); *, P < 0.05.

# Video S1. Signal communication between PB2 and zygote.

Nls-mCherry protein was injected into the zygote or PB2 at the early 1-cell stage. After injection, shutter of 594 nm laser was opened immediately to observe the position of Nls-mCherry protein. No matter where Nls-mCherry was injected, mCherry signal gradually appeared in the opposite side of the embryos, suggesting material exchange between PB2 and the zygote. Though the injected protein contains nuclear localization sequence, it took time for it to be enriched in the nucleus, so that we couldn’t observe stronger nucleus-localized mCherry signal in the short movie. The movie was taken under an Olympus IX73 inverted microscope and processed by Image J and Photoshop. The contrast of the movie was turned up to lower background signals.

# Video S2. Oil effectively labels pbB and npbB.

Olive oil was injected into PB side or NPB side of the zygote during 24-26 hpi at the 1- cell stage and the movie was taken from 26 hpi onward. Before the completion of the first cleavage, the oil droplet did not move inside the zygote. During the first cleavage, the position of PB2 was changed while the oil droplet remained stationary. If the oil was injected into the PB side, it stayed in pbB at the 2-cell stage; if it was injected into the NPB side, it stayed in npbB at the 2-cell stage. The movie was taken under a Nikon Ti2-E inverted microscope for 12 h and captured one picture every 4 min. Scale bar, 20 μm.

**Video S3. Animation of 3D-reconstituted blastocysts with labels.** At the 2-cell stage, npbB (left) or pbB (right) were injected with *nls-mCherry*. Blastocysts were immunostained for the trophectodermal marker Cdx2 together with nuclei staining by Hoechst33342. Nuclei of blastocysts were reconstructed using Imaris. The inner cells were determined from their positions and labels by viewing from different angles (see also Fig. 3).

**Video S4. Live imaging of embryos with pbB descendants labeled by Nls-mCherry.** *Caax-gfp* mRNA was injected into the zygote at the 1-cell stage to label the cytoplasm membrane, followed by oil injection into the NPB side. Then at late 2-cell stage, pbB was labeled by injecting with *nls-mCherry* mRNA to label nuclei of its descendants. Embryos were observed under an Olympus FV3000 confocal microscope at the interval of 15 min and the movie was processed by Imaris. Two time windows were included: the period from 8-cell to 16-cell stage and the period from 16-cell to 32-cell stage. The inner cells were determined by viewing from different focal planes (see also Fig. S8).

# Video S5. Process of PB2 removal.

PB2 removal at the 1-cell stage was performed between 24-27 hpi and 2-cell stage PB2 removal was performed between 42-45 hpi. The first step was to create a cleft in the ZP. We used a thin needle to penetrate the ZP near PB2, rubbing it on the surface of the embryo holder. After the embryo dropped from the thin needle, a cleft could be observed on ZP. Next, a thick needle, whose diameter was smaller than PB, was put into the embryos through the cleft in the ZP and the PB2 was sucked into the needle

and pulled out gently. The movie was taken under an Olympus IX73 inverted microscope and processed by Photoshop.
